# Structural and Spectroscopic Properties of Magnolol and Honokiol–Experimental and Theoretical Studies

**DOI:** 10.3390/ijms26136085

**Published:** 2025-06-25

**Authors:** Jacek Kujawski, Beata Drabińska, Katarzyna Dettlaff, Marcin Skotnicki, Agata Olszewska, Tomasz Ratajczak, Marianna Napierała, Marcin K. Chmielewski, Milena Kasprzak, Radosław Kujawski, Aleksandra Gostyńska-Stawna, Maciej Stawny

**Affiliations:** 1 Department of Organic Chemistry, Faculty of Pharmacy, Poznan University of Medical Sciences, Rokietnicka 3 Str., 60-806 Poznań, Poland; bdrabinska@yahoo.com (B.D.); marianna.h1030@gmail.com (M.N.); milena.kasprzak01@gmail.com (M.K.); 2 Department of Pharmaceutical Chemistry, Faculty of Pharmacy, Poznan University of Medical Sciences, Rokietnicka 3 Str., 60-806 Poznań, Poland; dettlaff@ump.edu.pl (K.D.); agostynska@ump.edu.pl (A.G.-S.); mstawny@ump.edu.pl (M.S.); 3 Department of Pharmaceutical Technology, Industrial Pharmacy Division, Faculty of Pharmacy, Poznan University of Medical Sciences, Rokietnicka 3 Str., 60-806 Poznań, Poland; marcskot@ump.edu.pl (M.S.);; 4 Liquid Dosage Form Laboratory, Research and Development Department, Pharmaceutical Works Polpharma S.A., Barska 31, 02-315 Warsaw, Poland; tomasz.r.ratajczak@gmail.com; 5 Institute of Bioorganic Chemistry, Polish Academy of Sciences, Noskowskiego 12/14 Str., 61-704 Poznań, Poland; chmielewskimk@ibch.poznan.pl; 6 Department of Pharmacology, Faculty of Pharmacy, Poznan University of Medical Sciences, Rokietnicka 3 Str., 60-806 Poznań, Poland; radkuj@ump.edu.pl

**Keywords:** density functional theory, natural bond orbital, nuclear magnetic resonance, magnolol, honokiol

## Abstract

This study presents an integrated experimental and theoretical investigation of two pharmacologically significant neolignans—magnolol and honokiol—with the aim of characterizing their structural and spectroscopic properties in detail. Experimental Fourier-transform infrared (FT-IR), ultraviolet–visible (UV-Vis), and nuclear magnetic resonance (^1^H NMR) spectra were recorded and analyzed. To support and interpret these findings, a series of density functional theory (DFT) and time-dependent DFT (TD-DFT) calculations were conducted using several hybrid and long-range corrected functionals (B3LYP, CAM-B3LYP, M06X, PW6B95D3, and ωB97XD). Implicit solvation effects were modeled using the CPCM approach across a variety of solvents. The theoretical spectra were systematically compared to experimental data to determine the most reliable computational approaches. Additionally, natural bond orbital (NBO) analysis, molecular electrostatic potential (MEP) mapping, and frontier molecular orbital (FMO) visualization were performed to explore electronic properties and reactivity descriptors. The results provide valuable insight into the structure–spectrum relationships of magnolol and honokiol and establish a computational benchmark for further studies on neolignan analogues.

## 1. Introduction

The pivotal role of natural bioactive products in pharmaceutical discovery and development is widely acknowledged. Such compounds have historically constituted, and continue to provide, a critical source for identifying novel therapeutic and diagnostic agents. Consequently, a significant percentage of small-molecule drugs currently employed in clinical practice originate directly from natural sources or are synthetic analogues based on natural product scaffolds [1,2].

Magnolol (**1**) and honokiol (**2**), natural bioactive compounds isolated from the bark of Magnolia officinalis, are presently undergoing intensive investigation for their therapeutic potential. They are classified as neolignans, a class of compounds characterized by the presence of two phenylpropanoid monomers within their molecular framework. These two structural isomers possess two hydroxyl moieties, the precise spatial arrangement of which critically dictates their biological properties and modulates their stability [3]. Historically, in traditional Chinese medicine, **1** and **2** were utilized for the management of thrombotic stroke, gastrointestinal disturbances, anxiety, nervous system disorders, and allergic and inflammatory diseases, as well as malignant neoplasms [4,5]. Contemporary studies have corroborated these uses and revealed additional biological activities, including antibacterial, antiviral, neuroprotective, hepatoprotective, cardioprotective, and antidiabetic properties [4,6]. Both **1** and **2** exhibit considerable potential to modulate a diverse array of intracellular signaling molecules and pathways crucial for oncogenesis [7,8]. Magnolol demonstrates considerable promise in reproductive system disorders, particularly through the amelioration of the polycystic ovary syndrome (PCOS) phenotype via the regulation of sex hormones and ovarian signaling pathways [9]. Honokiol may offer significant protection against the onset and progression of neurodegenerative diseases in humans, improving cognitive function and overall brain health [10].

In silico studies on the structural and spectroscopic analysis of **1** and **2** are notably scarce. Existing research primarily focuses on evaluating their antioxidant properties [11,12,13] or on studying their interactions with selected biological targets, such as the MoLAC14 enzyme [14], the COX-2 protein [15], or their anticancer effects against various cancer types [16]. To fill this gap, we compared the experimental FT-IR and UV-Vis spectra of **1** and **2** with theoretical data obtained using different basis sets and computational methods. Our goal was to determine which basis sets or methods yield the best agreement between theoretical predictions and experimental results. ^1^H NMR spectroscopy plays a crucial role in confirming the identity and purity of small organic molecules. Given the limited utility of ^13^C NMR for investigating small-molecule interactions, as well as the low natural abundance of ^13^C, we focused our analysis on ^1^H NMR spectroscopy [17,18]. Moreover, we aimed to identify the preferred conformations of these compounds from a quantum chemical perspective. This work may contribute to a better understanding of the structural and spectral features underpinning these molecules’ biological activity. The approximations employed in this study have previously been successfully applied by our team in spectral analyses, including those related to antifungal conazoles [19,20].

## 2. Results and Discussion

### 2.1. Geometry Optimization

**Figure 1:**
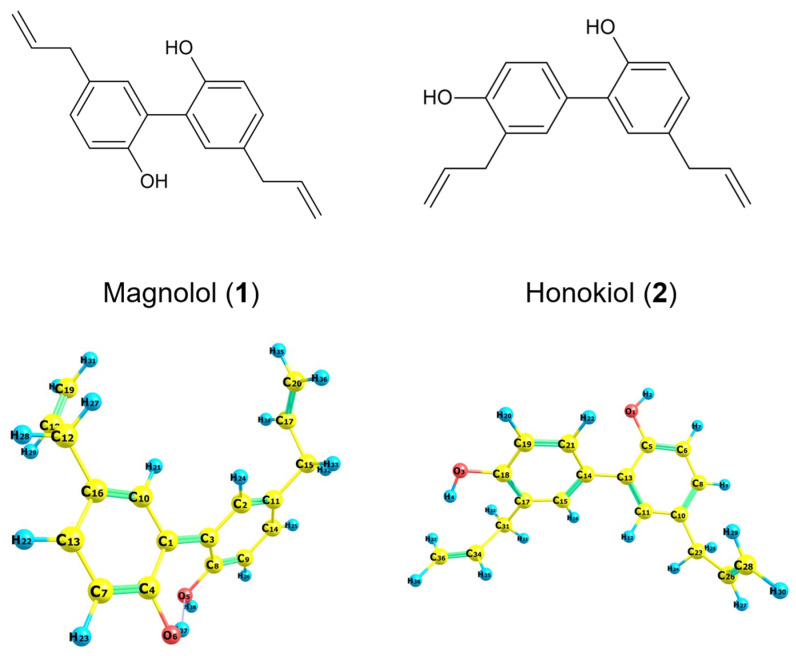
Formulas for magnolol (**left**) and honokiol (**right**) and their optimized geometry at the B3LYP/6-311+G(d,p) level of theory.

Considering the initial geometries of the rotamers **1** and **2** (formulas are given in the Figure 1), the magnitude of the RMSD error for the geometry of remaining optimized rotamers was 0.5074 and 0.4772 (for rotamers optimized at the B3LYP/6-31G(d,p) level of theory), 0.4482 and 0.4416 (for rotamers optimized at the B3LYP/6-311+G(d,p) level of theory), 0.5405 and 0.4787 (CAM-B3LYP/6-31G(d,p)), 0.5645 and 0.4895 (PBE1PBE/6-31G(d,p)), 0.6005 and 0.5422 (PW6B95D3/6-31G(d,p)), 0.6302 and 0.5252 (M06L/6-31G(d,p)), 0.5696 and 0.4850 (M062X/6-31G(d,p)), 0.5534 and 0.4830 (APF/6-31G(d,p)), 0.5713 and 0.4830 Å (APFD/6-31G(d,p)), respectively. It is, therefore, shown that the smallest RMSD value for **1** and **2** was obtained with respect to the B3LYP/6-311+G(d,p) approximation. Next, the molecular electrostatic potential (MEP) was determined by the B3LYP/6-311++G(2d,3p) approach for the selected compounds, with geometry previously optimized in the gaseous phase.

To the best of our knowledge, the studies regarding the charge analysis for analytes only involved M06-2X functional with 6-31+G* basis set or B3LYP functional and the 6-31G(d,p) basis set and concerned their antioxidant activity [12] or their derivatives [21]. In our investigations, involving the multilevel approach to the conformational rotamers search, the results were refined using a basis set enriched with the higher-level polarization functions.

**Figure 2:**
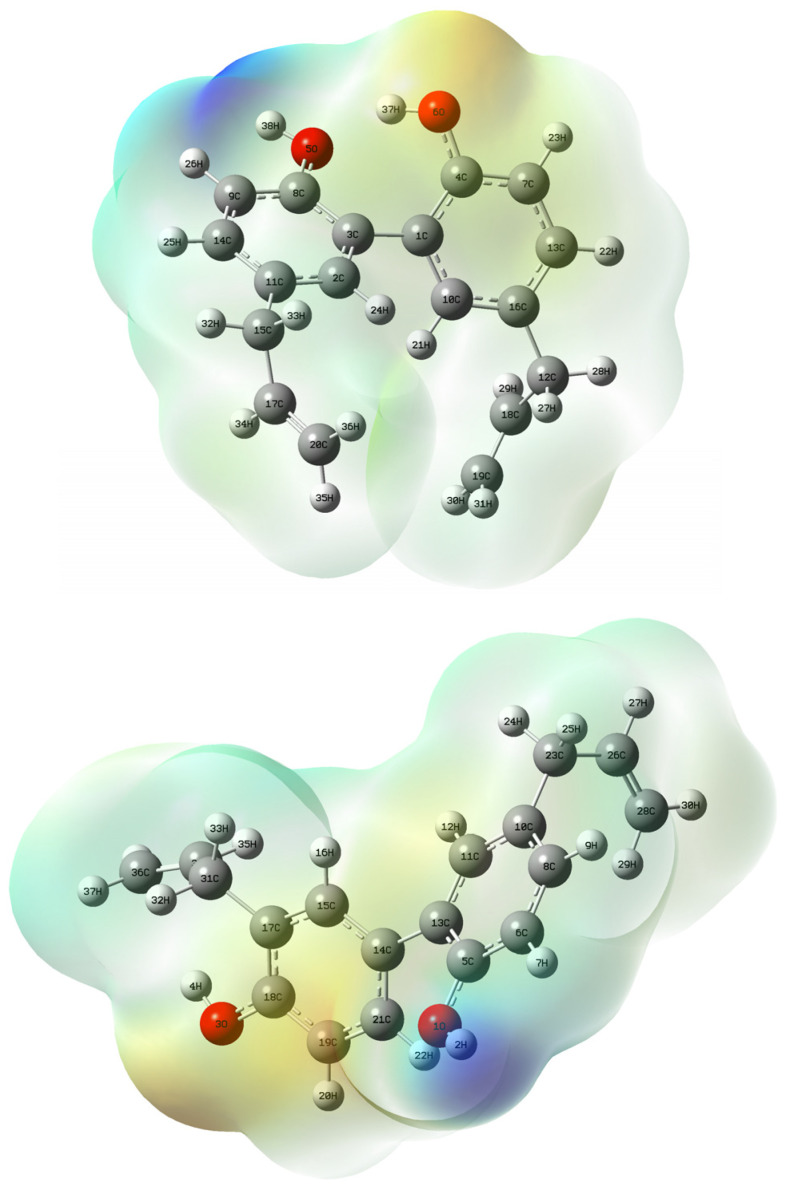
Electrostatic potential (ESP) map of magnolol **1** (**up**) and honokiol **2** (**down**) calculated at the B3LYP/6-311++G(2d,3p)//B3LYP-6311++G(d,p) level of theory; gaseous phase; isovalue = 0.0004 a.u.; scale: red–blue from −6.959 × 10^−2^ to −6.959 × ^−2^.

The list of atomic charges estimated according to the Mulliken, CHelpG [22], and natural bond orbital (NBO) methodologies is given in Table S1 (given in the Supplementary Material). We compared atomic charges obtained from Mulliken population analysis and NBO analysis to interpret the electron density distribution on the oxygen atoms in magnolol and honokiol. Notably, the Mulliken method yielded substantial variability in computed oxygen charges—absolute differences of 0.114 (oxygen **1**) and 0.064 (oxygen **2**)—across the two compounds. In contrast, NBO consistently produced highly negative and chemically intuitive oxygen charges with minimal variation between the molecules. This behavior can be attributed to the fundamental differences in how these charge schemes partition electron density (MEP, Figure 2). Mulliken charges rely on an arbitrary allocation of electron density based on orbital overlaps, making them highly sensitive to the choice and completeness of the basis set. This sensitivity leads to unstable and sometimes unphysical values, especially for electronegative atoms in delocalized systems such as phenols. On the other hand, NBO analysis employs a wavefunction-driven localization procedure, assigning electron density into Lewis-type bonding, antibonding, and lone-pair orbitals. This enables it to accurately represent non-bonding lone pairs on oxygen atoms, yielding more negative and consistent charges, particularly in phenolic OH groups where the lone-pair character is pronounced [23,24]. These results validate the superiority of NBO over Mulliken analysis for assessing partial charges in phenolic systems such as magnolol and honokiol and justify its preferential use in the current study.

### 2.2. IR Analysis

**Figure 3:**
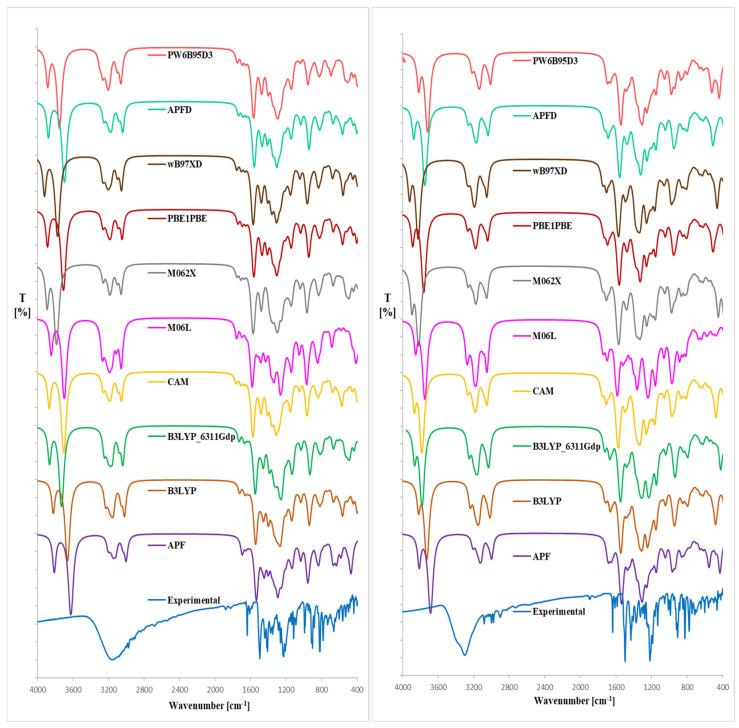
Expended experimental (**EXP**) and theoretical IR spectra (DFT formalism, gaseous phase) of magnolol (**left**) or honokiol (**right**); **B3LYP**-B3LYP/6-31G(d,p)//B3LYP/6-31G(d,p) approach, **B3LYP_6311Gdp**–B3LYP/6-311++G(d,p)//B3LYP/6-311++G(d,p) approach, **CAM**–CAM-B3LYP/6-31G(d,p)//CAM-B3LYP/6-31G(d,p) approach, **M06L**–M06L/6-31G(d,p)//M06L/6-31G(d,p) approach, **M062X**–M062X/6-31G(d,p)//M062X/6-31G(d,p) approach, **APF**–APF/6-31G(d,p)//APF/6-31G(d,p) approach, **APFD**–APFD/6-31G(d,p)//APF/6-31G(d,p) approach, **PBE1PBE**–PBE1PBE/6-31G(d,p)//PBE1PBE/6-31G(d,p) approach, **PW6B95D3**–PW6B95D3/6-31G(d,p)//PW6B95D3/6-31G(d,p) approach.

For the theoretical analysis of magnolol (**1**) and honokiol (**2**) IR (Table 1 and Table 2) we carried out the computations of **1** and **2** vibrational frequencies using B3LYP, CAM-B3LYP, PB0, M06L, M062X, PW6B95D3 functionals [25,26,27], and the resulted IR spectra are shown in Figure 3. Small differences between the experimental and calculated vibrational modes can be observed because the experimental results were obtained in the solid phase whereas the theoretical calculations were carried out in the gaseous phase.

To the best of our knowledge, the literature does not report theoretical IR spectra of **1** and **2** calculated using the DFT formalism. Given that the lowest RMSD relative to the crystal structures of **1** and **2** was obtained using the B3LYP/6-311++G(d,p)//B3LYP/6-31G(d,p) approximation, we applied the same computational approach for the theoretical IR spectra of the analytes.

From the spectra given in Figure 3, we can conclude that the use of the B3LYP/6-311++G(d,p) approach for the optimized rotamers gives the highest conformity of the theoretical IR bands with the experimental spectrum, particularly below ca. 3120 (**1**) or 3260 cm^−1^ (**2**). The utilization of Grimme’s D3 empirical dispersion model did not lead to better consistency between the theoretical and experimental IR spectral values. Moreover, including the approximation of B3LYP/6-31G(d,p) did not lead to better consistency of the theoretical IR spectrum comparable with the experimental one (as it is relatively less time-consuming in comparison with the B3LYP/6-311++G(d,p) functional). The OH stretching bands were visible at ca. 3860 (**1**) and 3900 cm^−1^ (**2**), thus at the highest wavenumbers than for the experimental spectrum. In the computed spectra of **1** and **2**, the estimated υ C-C, υ C=C, υ C-H, υ C-O, δ C-H, and γ C-H (related with benzene rings) absorptions were in excellent accordance with the experimental and literature data [13,28,29,30,31,32]. Regarding the theoretical IR results (Figure 3, Table 1 and Table 2) in the 3200–4000 cm^−1^, the discrepancy arises due to anharmonic effects and limitations in reproducing hydrogen-bonded OH stretches using harmonic DFT, and the exchangeable nature of the OH proton. Such high-frequency shifts are typical of B3LYP and other hybrid functionals without anharmonic corrections. It turned out that the use of the B3LYP functional seems to be comparatively more effective because these approaches generally afford results without significant errors. A similar conclusion can be drawn from the method involving other functionals used in our investigations.

### 2.3. UV-Vis Analysis

**Figure 4:**
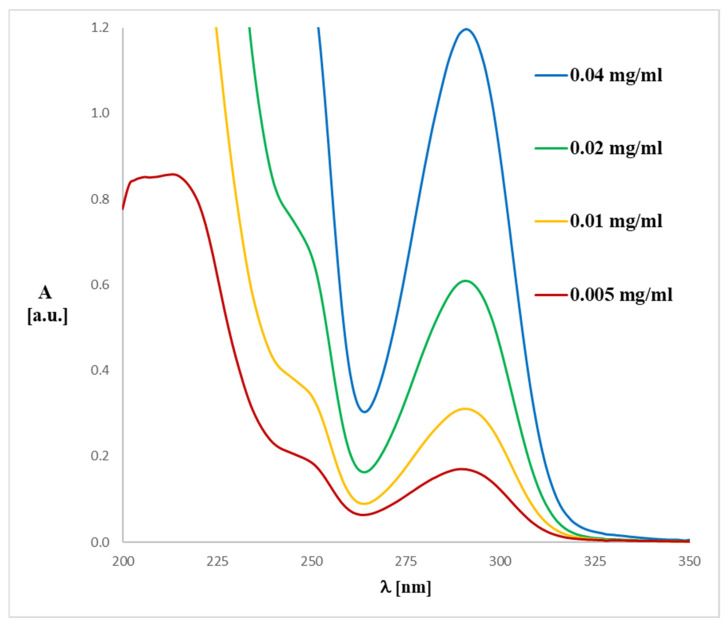
Experimental UV-Vis spectrum of magnolol (**1**) registered in methanol at various concentrations.

**Figure 5:**
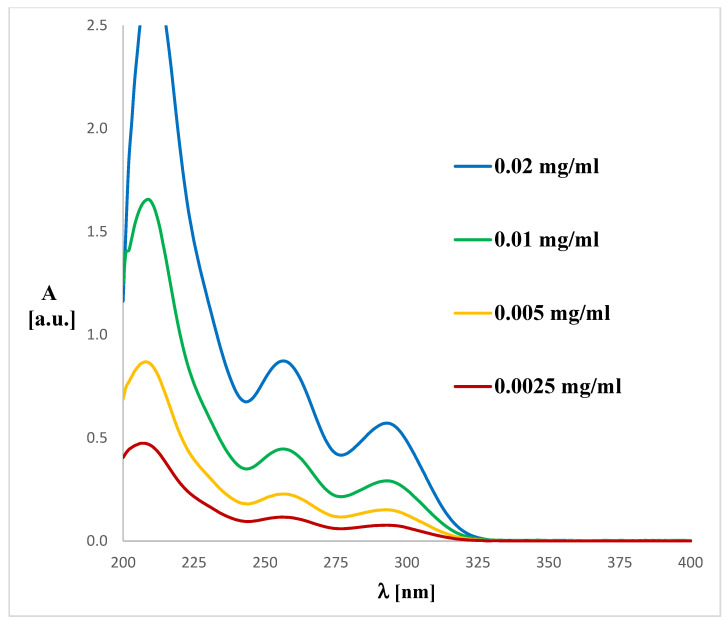
Experimental UV-Vis spectrum of honokiol (**2**) registered in methanol at various concentrations.

The UV spectrum for **1** and **2** is in accordance with the literature data [33]. The spectra (Figure 4 and Figure 5) display an absorption band at 290–291 nm (λ_max_), which did not change with the concentration used. However, another absorption bands of analytes **1** or **2** are observed at ca. 241–258 nm, which migrates as a function of concentration.

**Figure 6:**
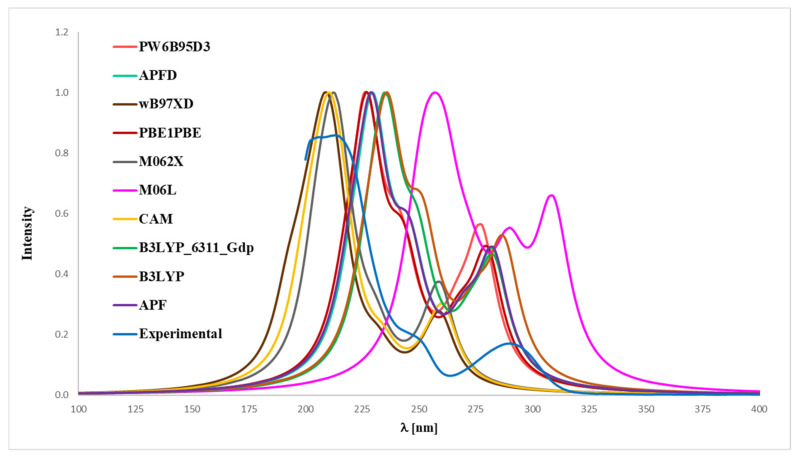
Experimental (EXP) and theoretical UV-Vis spectra of magnolol (**1**) registered in methanol and computed using CPCM solvation model (methanol as solvent); approximations: **APF**–APF/6-311++G(2d,3p)//APF/6-31G(d,p), **B3LYP**–B3LYP/6-311++G(2d,3p)//B3LYP/6-31G(d,p), **APFD**–APFD/6-311++G(2d,3p)//APFD/6-31G(d,p), **CAM**–CAM-B3LYP/6-311++G(2d,3p)//CAM-B3LYP/6-31G(d,p), **M06L**–M06L/6-311++G(2d,3p)//M06L/6-31G(d,p), **PBE1PBE**–PBE1PBE/6-311++G(2d,3p)//PBE1PBE/6-31G(d,p), **PW6B95D3**–PW6B95D3/6-311++G(2d,3p)//PW6B95D3/6-31G(d,p), **M062X**–M062X/6-311++G(2d,3p)//M062X/6-31G(d,p), **B3LYP_6311_Gdp–**B3LYP/6-311++G(2d,3p)//B3LYP/6-311++G(d,p), **wB97XD**–wB97XD/6-311++G(2d,3p)//wB97XD/6-31G(d,p).

**Figure 7:**
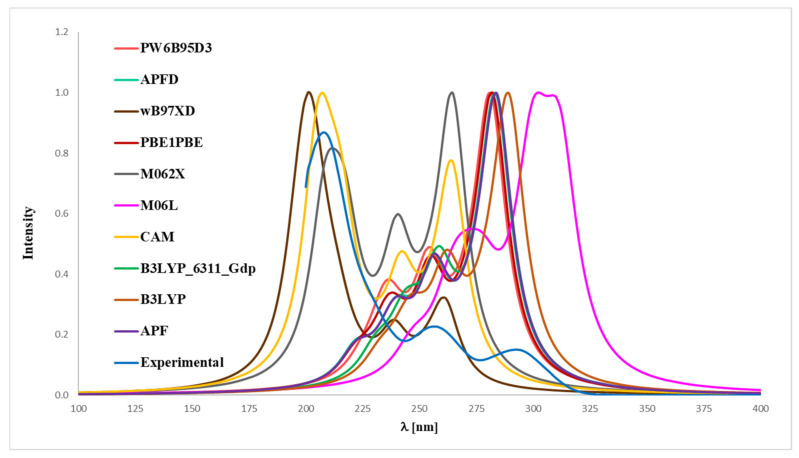
Experimental (EXP) and theoretical UV-Vis spectra of honokiol (**2**) registered in methanol and computed using CPCM solvation model (methanol as solvent); approximations: **APF**–APF/6-311++G(2d,3p)//APF/6-31G(d,p), **B3LYP**–B3LYP/6-311++G(2d,3p)//B3LYP/6-31G(d,p), **APFD**–APFD/6-311++G(2d,3p)//APFD/6-31G(d,p), **CAM**–CAM-B3LYP/6-311++G(2d,3p)//CAM-B3LYP/6-31G(d,p), **M06L**–M06L/6-311++G(2d,3p)//M06L/6-31G(d,p), **PBE1PBE**–PBE1PBE/6-311++G(2d,3p)//PBE1PBE/6-31G(d,p), **PW6B95D3**–PW6B95D3/6-311++G(2d,3p)//PW6B95D3/6-31G(d,p), **M062X**–M062X/6-311++G(2d,3p)//M062X/6-31G(d,p), **B3LYP_6311_Gdp–**B3LYP/6-311++G(2d,3p)//B3LYP/6-311++G(d,p), **wB97XD**–wB97XD/6-311++G(2d,3p)//wB97XD/6-31G(d,p).

To match the experimental (Figure 4 and Figure 5) and theoretical UV-Vis spectra of analytes **1** and **2** (Figure 6 and Figure 7), we optimized the molecules’ geometry and applied the linear response time-dependent DFT (TD-DFT) method for the calculations. The vertical excited states were calculated for each optimized rotamer of compounds **1** and **2** at the functional/6-311++G(2d,3p) level of theory in the gas phase, as well as in methanol (CPCM solvation model).

In the case of magnolol **1** (Figure 6), the correspondence to the experimental data, especially with reference to the 291 nm band, was obtained using the B3LYP/6-31++G(d,p) approach (absolute value of Δ = 2.17 nm), B3LYP (absolute value of Δ = 3.80 nm), APFD (absolute value of Δ = 8.47 nm), APF (absolute value of Δ = 8.57 nm), PBE1PBE (absolute value of Δ = 11.02 nm), PW6B95D3 (absolute value of Δ = 13.57 nm), M06L (absolute value of Δ = 17.95 nm), CAM-B3LYP (absolute value of Δ = 30.19 nm), wB97XD (absolute value of Δ = 31.51 nm) and M062X functionals (absolute value of Δ = 31.89 nm). Whereas in the case of honokiol **2** (Figure 7), with a reference to the experimental band 291 nm, the agreement could be achieved using the B3LYP functional (absolute value of Δ = 1.95 nm), B3LYP/^-31++G(d,p) approach (absolute value of Δ = 7.15 nm), APF (absolute value of Δ = 7.36 nm), APFD (absolute value of Δ = 7.67 nm), M06L (absolute value of Δ = 8.74 nm), PBE1PBE (absolute value of Δ = 9.17 nm), PW6B95D3 (absolute value of Δ = 10.03 nm), M062X (absolute value of Δ = 26.73 nm), CAM-B3LYP (absolute value of Δ = 26.91 nm) and additionally the wB97XD functionals (absolute value of Δ = 29.82 nm). It turned out that, for compound **2**, none of the tested functionals reproduced the experimental distribution of signals in the theoretical UV-Vis spectrum, except for the wB97XD functional. Specifically, the maximum-intensity signal predicted by other functionals appeared at the most bathochromically shifted position, in contrast to the 291 nm band observed in the experimental spectrum. Thus, it can be concluded that the application of the wB97XD functional represents a favorable approach for accurately predicting the UV-Vis spectrum of **2**.

The results of calculations involving the first excited states of **1** and **2** are collected in Tables S2 (B3LYP functional) and S3 (wB97XD functional) given in the Supplementary Material.

**Figure 8:**
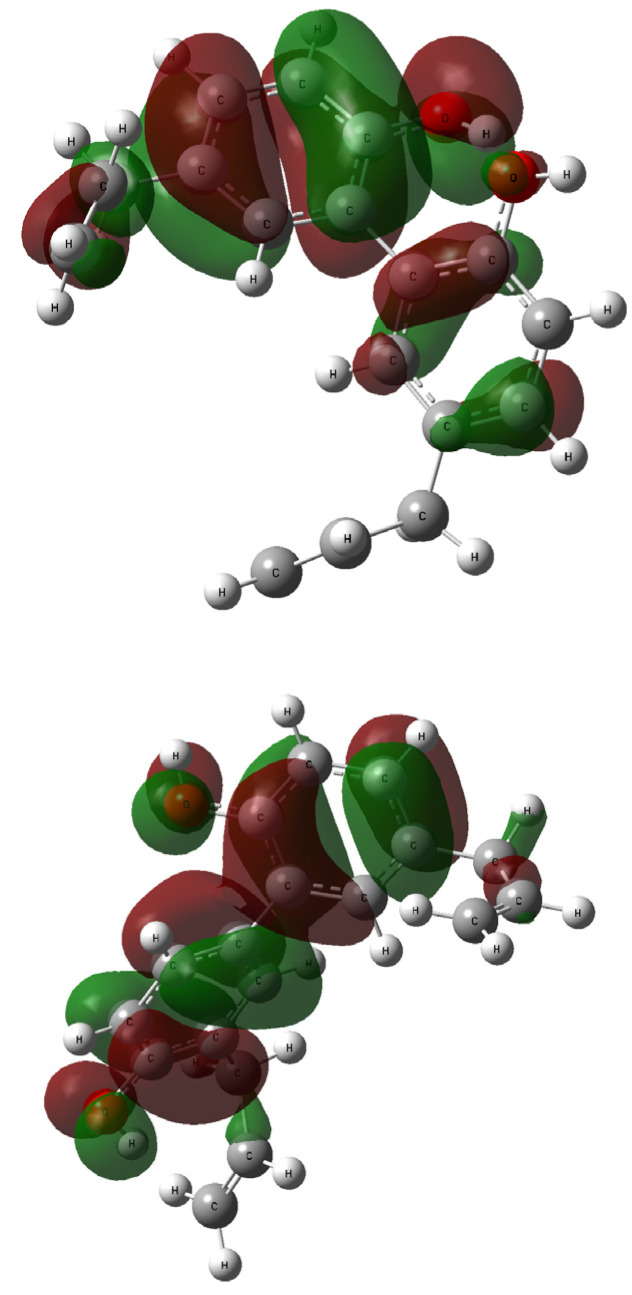
The HOMO orbitals generated for compound **1** (**up**: rotamer optimized at the B3LYP /6-311++G(d,p) level of theory in methanol) and **2** (**down**: rotamer optimized at the wB97XD/6-31G(d,p) level of theory in methanol); vertical excited states calculated at the B3LYP/6-311++G(2d,3p) (**1**) or wB97XD/6-311++G(2d,3p) (**2**) levels of theory.

**Figure 9:**
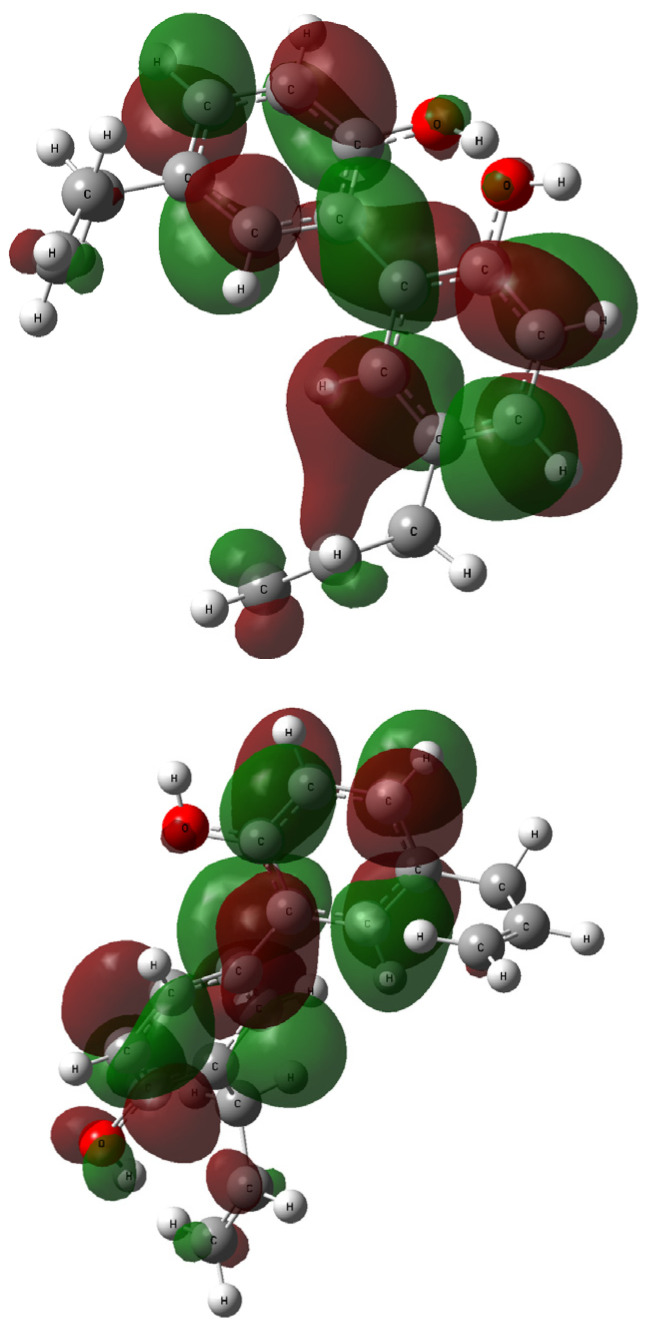
The LUMO orbitals generated for compound **1** (**up**: rotamer optimized at the B3LYP/6-311++G(d,p) level of theory in methanol) and **2** (**down**: rotamer optimized at the wB97XD/6-31G(d,p) level of theory in methanol); vertical excited states calculated at the B3LYP/6-311++G(2d,3p) (**1**) or wB97XD/6-311++G(2d,3p) (**2**) levels of theory.

The contours of the LUMO and HOMO orbitals for **1** and **2**—generated during the TD-DFT computations—are presented in Figure 8 and Figure 9, respectively. The highest occupied molecular orbital (HOMO) is located mainly over all biphenyl rings, and the hydroxyl moieties linker. The similarities between **1** and **2** are also related to the lowest occupied molecular orbitals (LUMOs) and their coverage of the biphenyl residue.

The absolute value of the HOMO–LUMO gap calculated for magnolol **1** at the B3LYP/6-311++G(2d,3p) level is 5.0137 eV corresponding to an electron transition from spinorbital 71 to spinorbital 72. It can be assigned to the calculated first excitation state at 282.79 nm and is higher than for honokiol **2** where that gap was estimated at 4.9509 eV [B3LYP/6-311++G(2d,3p)//B3LYP/6-311++G(d,p) approach]. On the other hand, the HOMO–LUMO gap calculated for **2** at the wB97XD/6-311++G(2d,3p) level is 8.6321 eV is related to an electron transition from spinorbital 71 to spinorbital 72 and the first excitation state at 261.18 nm and is slightly lower than for magnolol **1** where that gap was estimated at 8.7205 eV [wB97XD/6-311++G(2d,3p)//wB97XD/6-31G(d,p) approach].

The first excited state for compound **1** [B3LYP/6-311++G(2d,3p)//B3LYP/6-311++G(d,p) approach] relates mainly to a HOMO → LUMO transition (oscillator strength f = 0.2052, coefficient 0.67505, calculated energy is 4.3844 eV; data taken from the output file). In this case, the HOMO−LUMO contribution is 91%, and is the same as contribution for honokiol **2** [91%, 261.18 nm, HOMO → LUMO transition, oscillator strength f = 0.3701, coefficient 0.66748, calculated energy is 4.3680 eV, B3LYP/6-311++G(2d,3p)//B3LYP/6-311++G(d,p) approach]. In the case of the UV spectrum of honokiol **2** computed using the wB97XD/6-31G(2d,3p)//wB97XD/6-31G(d,p) approximation the first excited state is described by the following parameters: electron excitation from spinorbital 71 to spinorbital 72 and a HOMO → LUMO transition (oscillator strength f = 0.3402, coefficient 0.58692, calculated energy is 4.7471 eV) is 69%%, and is lower than the contribution for magnolol **1** [75%, 259.49 nm, HOMO → LUMO transition, oscillator strength f = 0.2558, coefficient 0.61098, calculated energy is 4.7780 eV, wB97XD/6-31G(2d,3p)//wB97XD/6-31G(d,p) approach]. It is worth mentioning that in the theoretical UV-Vis spectra of **1** and **2**, the highest oscillator strength can be assigned to the third excitation state. The above discussion shows that the DFT method can satisfactorily explain the observations taken from the experimental UV-Vis spectra of the analyzed compounds.

Next, for **1** and **2** we computed several descriptors related to HOMO–LUMO electron transition, i.e., first ionization potential (I), electron affinity (A), electronegativity (χ), chemical hardness (η), electronic potential (μ) using the orbital energy of the HOMO and the orbital energy of the LUMO based on the DFT formalism, as well as the chemical potential of the molecule using Koopman’s theorem [34,35]. Regarding the above-mentioned data, the results are given in Table 3 [eV].

It is worth mentioning that previous literature reports on HOMO–LUMO descriptors for magnolol and honokiol were limited to calculations using the B3LYP functional at the 6-31G* basis set level in vacuo [31]. The HOMO energy value (B3LYP functional) obtained for magnolol (**1**) in our study is consistent with the literature data [31]. However, across all functionals used in our work, the HOMO energy of magnolol (**1**) consistently exhibited more negative values compared to the HOMO energy of honokiol (**2**). Similarly, the LUMO energy of magnolol (**1**), calculated with all tested functionals, was consistently more negative than that of honokiol (**2**) (Table 3). As a result, the absolute values of χ (electronegativity), η (chemical hardness), and μ (chemical potential) were greater for magnolol (**1**) across all applied functionals.

**Figure 10:**
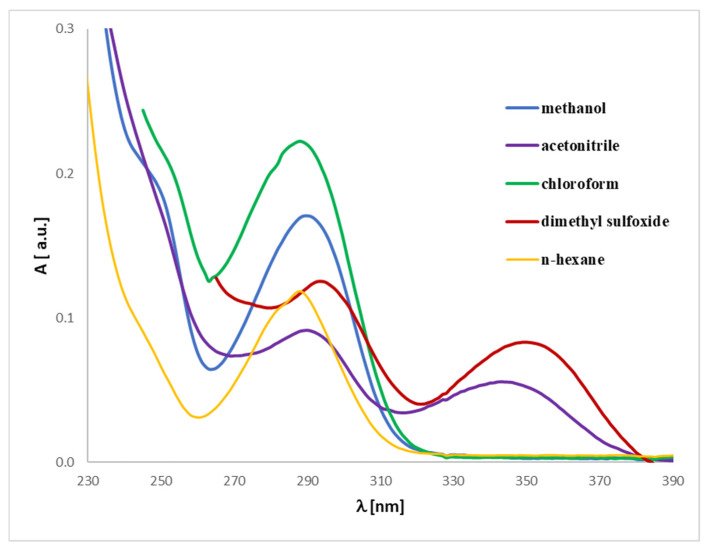
Experimental UV-Vis spectra of magnolol (**1**) registered in different solvents at a concentration of 0.005 mg/mL.

**Figure 11:**
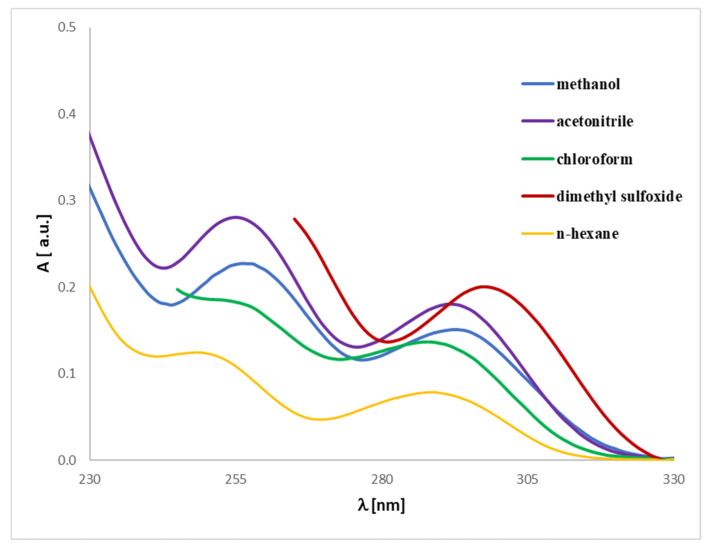
Experimental UV-Vis spectra of honokiol (**2**) registered in different solvents at a concentration of 0.005 mg/mL.

The UV-Vis analysis for magnolol or honokiol in various solvents (methanol, acetonitrile, chloroform, dimethylsulfoxide, and *n*-hexane) can be interpreted similarly to the prior analysis of discussed analytes (Figure 10 and Figure 11). Due to their strong absorption in the lower UV range, DMSO and chloroform exhibit significant spectral limitations in UV-Vis measurements. The cutoff wavelength, below which a solvent begins to absorb UV radiation strongly, is approximately 265 nm for DMSO and 245 nm for chloroform. As a result, the spectra recorded in these solvents are presented only above their respective cutoff wavelengths. The data reveal absorbance values for magnolol in different solvents, where notable spectral differences are visible. These differences arise from the unique interactions between magnolol and the solvents, driven by their polarity and solvent properties. By examining the absorbance maxima (λ_max_), we can determine the bathochromic (red shift) or hypsochromic (blue shift) shifts due to solvent polarity. For example: in methanol, a protic solvent, magnolol **1** or honokiol **2** show an absorbance of 0.00215 (**1**) or 0.00231 (**2**) at 400 nm, and in *n*-hexane, a non-polar solvent, magnolol has a higher absorbance of 0.00401 (**1**) or 0.00394 (**2**) at the same wavelength. These trends suggest solvatochromism, where magnolol and honokiol interact differently with polar and non-polar solvents, shifting their absorption band. Bathochromic shifts (red shifts) are expected in non-polar solvents, like *n*-hexane, due to weaker solute-solvent interactions, leading to a shift toward higher wavelengths (lower energy). Hypsochromic shifts (blue shifts) are more likely in polar solvents, such as acetonitrile or dimethylsulfoxide (DMSO), which stabilize the excited state and shift absorption toward shorter wavelengths. Comparing the first excited state in various solvents would involve noting the absorbance peaks at the lower energy transitions (near the onset of absorbance). For instance, the methanol and acetonitrile data show lower absorbance in the lower wavelength range compared to *n*-hexane, reflecting hypochromic shifts in polar solvents. Methanol and DMSO, as polar solvents, show distinct interactions with magnolol, affecting the UV spectrum. The analytes show lower absorbance in these solvents, likely due to stronger hydrogen bonding or dipole-dipole interactions. In contrast, *n*-hexane, being non-polar, shows relatively higher absorbance values, indicating less solvent–solute interaction and, therefore, stronger absorption of UV light. The comparison across solvents highlights how polarity influences the analyte’s UV absorbance. Non-polar solvents like *n*-hexane shift the λ_max_ toward higher wavelengths, while polar solvents like methanol and acetonitrile shift it toward shorter wavelengths, consistent with solvatochromic behavior.

In conclusion, the UV-Vis spectra of magnolol or honokiol vary significantly based on the solvent used, driven by polarity and solute-solvent interactions. Bathochromic and hypsochromic shifts provide insight into these interactions, with non-polar solvents generally causing red shifts and polar solvents causing blue shifts. In addition, our findings significantly enrich the knowledge in this aspect, which, so far, is limited in the literature to considerations of the analytes.

### 2.4. NBO Analysis

Considering the conclusions drawn from the UV-Vis analysis, we carried out Natural Bond Orbitals (NBO) studies (CPCM solvation model and methanol used as solvent). The NBO analysis was performed at the wB97XD/6-311++G(2d,3p) level of theory using the *NBO 3.0* approach as implemented in *Gaussian G16 A.03* software for rotamers previously optimized in the wB97XD/6-31G(d,p) approximation. Our attention was focused on the oxygen atoms, as well as aromatic rings which electrons were important for the distribution of HOMO and LUMO orbitals (Figure 1). The second-order perturbation theory, which involves the Fock matrix in the NBO basis, shows intramolecular hyper-conjugative interactions.

The C1-C3 bond and the rings formed by C1, C4, C7, C13, C16, C10, C3, C2, C11, C14, C9, and C8 atoms in magnolol **1** (Figure 1) can be depicted by an almost completely filled (1.96690, 1.97121, 1.97489, 1.97443, 1.97292, 1.66163, 1.96480, 1.97147, 1.97323, 1.97776, 1.66539, and 1.96671*e* for C1-C3, C1-C4, C4-C7, C7-C13, C13-C16, C10-C16, C2-C3, C2-C11, C11-C14, C9-C14, C8-C9, and C3-C8 bonds, respectively) 2-centre bonding hybrid BD orbital (polarization coefficients: 0.7051, 0.7316, 0.7113, 0.7084, 0.7111, 0.7151, 0.7146, 0.7083, 0.7106, 0.7107, 0.7233, and 0.7092 for C1-C3, C1-C4, C4-C7, C7-C13, C13-C16, C10-C16, C2-C3, C2-C11, C11-C14, C9-C14, C8-C9, and C3-C8 bonds, respectively) formed by interaction between *s* (36.11, 0, 37.03, 36.64, 34.46, 0, 34.47, 33.99, 34.48, 36.69, 0, and 33.15% *s* for C1-C3, C1-C4, C4-C7, C7-C13, C13-C16, C10-C16, C2-C3, C2-C11, C11-C14, C9-C14, C8-C9, and C3-C8 bonds, respectively) and *p* (68.24% *p*^2.11^, 99.97% *p*^1.00^, 62.92% *p*^1.70^, 63.26% *p*^1.73^, 65.45% *p*^1.90^, 99.97% *p*^1.00^, 65.39% *p*^1.90^, 65.94% *p*^1.94^, 65.43% *p*^1.90^, 63.21% *p*^1.72^, 99.93% *p*^1.00^, and 66.73% *p*^2.01^ for C1-C3, C1-C4, C4-C7, C7-C13, C13-C16, C10-C16, C2-C3, C2-C11, C11-C14, C9-C14, C8-C9, and C3-C8 bonds, respectively) orbitals. The C3, C1, C4, C7, C16, C16, C3, C11, C11, C9, C9, and C3 carbon atoms have a greater contribution (50.28, 53.53, 50.59, 50.18, 50.57, 51.13, 51.07, 50.17, 50.50, 50.50, 52.31, and 50.30% for C1-C3, C1-C4, C4-C7, C7-C13, C13-C16, C10-C16, C2-C3, C2-C11, C11-C14, C9-C14, C8-C9, and C3-C8 bonds, respectively) to these σ_C-C_ bonding orbitals.

Similarly to magnolol **1**, the C13-C14 bond and the rings formed by C14, C15, C17, C18, C19, C13, C5, C6, C8, C10 and C11 atoms in honokiol **2** (Figure 1) can be depicted by an almost completely filled (1.96881, 1.96927, 1.97190, 1.97425, 1.97472, 1.61838, 1.97689, 1.97353, 1.97228, 1.97175, and 1.96614*e* for C14-C15, C15-C17, C17-C18, C18-C19, C19-C21, C5-C13, C5-C6, C6-C8, C8-C10, C10-C11, and C11-C13 bonds, respectively) 2-centre bonding hybrid BD orbital (polarization coefficients: 0.7131, 0.7145, 0.7081, 0.7121, 0.7089, 0.7287, 0.7027, 0.7100, 0.7099, 0.7084, and 0.7139 for C14-C15, C15-C17, C17-C18, C18-C19, C19-C21, C5-C13, C5-C6, C6-C8, C8-C10, C10-C11, and C11-C13 bonds, respectively) formed by interaction between *s* (33.95, 35.68, 32.89, 36.99, 36.73, 0, 38.02, 36.83, 34.09, 34.09, and 34.42 *s* for C14-C15, C15-C17, C17-C18, C18-C19, C19-C21, C5-C13, C5-C6, C6-C8, C8-C10, C10-C11, and C11-C13 bonds, respectively) and *p* (65.98% *p*^1.94^, 64.24% *p*^1.80^, 67.00% *p*^2.04^, 62.95% *p*^1.70^, 63.18% *p*^1.72^, 99.96% *p*^1.00^, 61.92% *p*^1.63^, 63.07% *p*^1.71^, 65.82% *p*^1.93^, 65.83% *p*^1.93^, and 65.44% *p*^1.90^ for C14-C15, C15-C17, C17-C18, C18-C19, C19-C21, C5-C13, C5-C6, C6-C8, C8-C10, C10-C11, and C11-C13 bonds, respectively) orbitals. The C4, C17, C17, C18, C19, C5, C5, C6, C10, C10, and C13 carbon atoms have a greater contribution (50.86, 51.06, 50.15, 50.70, 50.26, 53.10, 50.62, 50.41, 50.40, 50.19, and 50.97 for C14-C15, C15-C17, C17-C18, C18-C19, C19-C21, C5-C13, C5-C6, C6-C8, C8-C10, C10-C11, and C11-C13 bonds, respectively) to these σ_C-C_ bonding orbitals.

The above bonds in the rotamers of **1** and **2** are a NBO density donor to the many bonds formed by the antibonding orbitals BD*. These bonds can also interact with the antibonding Rydberg orbitals RY* of many carbon atoms.

The O6-H37 and O5-H38 bonds in compound **1** (Figure 1) or O1-H2 and O3-H4 bonds in compound **2** (Figure 1) can be characterized by an almost completely filled (1.98154 and 1.98930*e* for O6-H37 and O5-H38 bonds in **1**, respectively or 1.98801 and 1.98633*e* for O1-H2 and O3-H4 bonds in **2**, respectively) 2-centre hybrid bonding orbital (polarization coefficients: 0.8742 and 0.8693 for O6-H37 and O5-H38 bonds in **1**, respectively or 0.8649 and 0.8701 for O1-H2 and O3-H4 bonds in **2**, respectively) formed by the overlap of *s* (23.58 and 21.84% *s*), *p* (76.05% *p*^3.22^ and 77.83% *p*^3.22^) orbitals for O6-H37 and O5-H38 bonds in **1**, respectively or *s* (21.25 and 23.31% *s*), *p* (78.39% *p*^3.22^ and 76.34% *p*^3.27^) for O6-H37 and O5-H38 bonds in **2**, respectively.

The oxygen atom has a greater contribution (76.43 and 75.56% for O6-H37 and O5-H38 bonds in **1**, respectively or 74.81 and 75.71% for O6-H37 and O5-H38 bonds in **2**, respectively) to the formation of this σ_O-H_ bonding orbital. These bonds are also NBO density donors to the following bonds formed by the antibonding orbital BD*, as well as antibonding Rydberg orbitals RY*.

The fundamental similarity of the NBO analysis between magnolol **1** and honokiol **2** are due to the presence of the biphenyl system with hydroxyl moieties within the structure of the analytes.

Considering the above data, we can conclude that the distribution of the NBOs for rotamers of magnolol and honokiol is almost identical and especially covers the biphenyl core.

### 2.5. NMR Analysis

**Figure 12:**
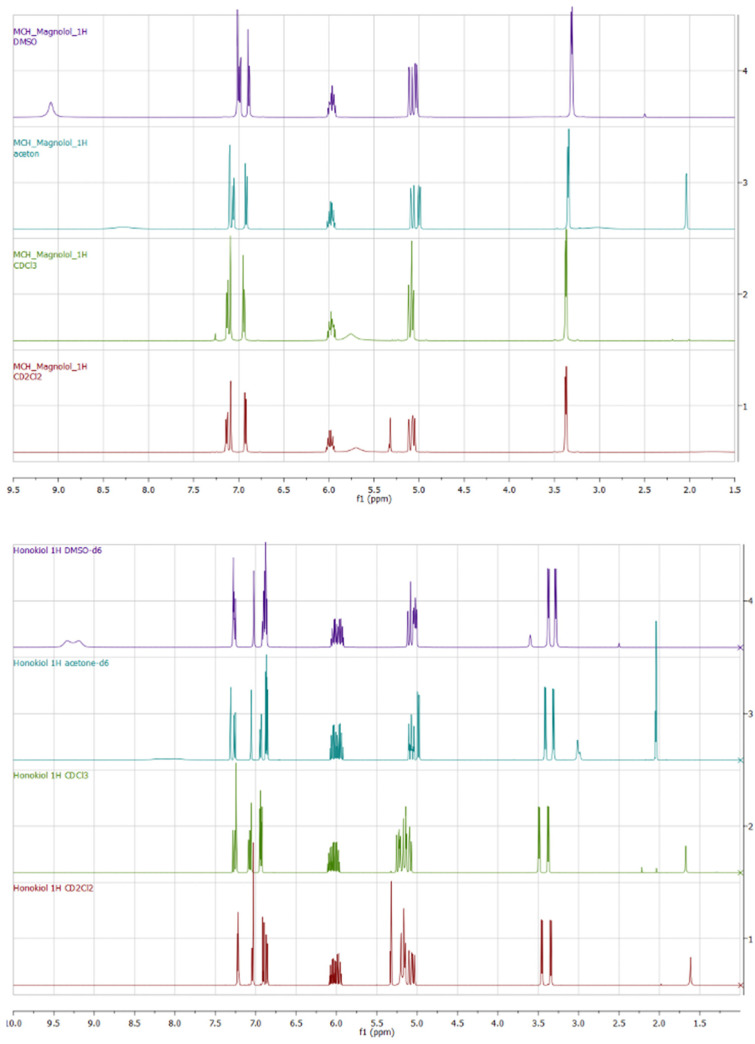
Experimental ^1^H NMR spectra of **1** (up) and **2** (down) registered in different solvents.

Magnolol and honokiol belong to the constitutive isomers. Magnolol possesses a plane of symmetry, while honokiol does not. Chemical shifts of compounds **1** and **2** can be compared in terms of different solvents. All ^1^H spectra, especially in aliphatic regions, can be differentiated and correlate well with the dielectric constant of the solvent. In general, the higher the dielectric constant (DMSO-d_6_ > acetone-d_6_ > CDCl_3_ > CD_2_Cl_2_), the more shifted upfield are the signals (Figure 12).

The OH groups in **1** and **2** are clearly visible only in DMSO-d_6_ (Table 4), giving broad signals, while exchange kinetics in CD_2_Cl_2_, CDCl_3_, and acetone-d_6_ rapidly show OH signals in the spectra. The magnolol 1D ^1^H spectrum is characterized by a singlet at 9.08 ppm, while two singlets in honokiol are downshifted to 9.19 and 9.33 ppm.

Magnolol’s protons 22-H are symmetric to 25-H, giving a doublet of doublets, and these signals are downshifted contrary to honokiol’s 20-H (doublet with *J* = 8.2 Hz). Honokiol’s 22-H at 7.24–7.26 ppm (dd, *J =* 2.2, 8.2 Hz, 1H) can be compared to magnolol’s proton 23-H (and symmetric 26-H) and is downshifted by around 0.36 ppm (Figure S1 given in the Supplementary Material). The coupling constant of honokiol between 20-H and 22-H (*J* = 8.2 Hz) is much larger than that between 22-H and 16-H (*J* = 2.1 Hz). The HMBC spectrum of magnolol does not allow us to distinguish between 23-H (26-H) and 21-H (24-H) protons.

Compounds **1** and **2** can be compared in terms of the shift of allyl groups. Magnolol, due to its symmetry, is characterized by one group of protons corresponding to CH_2_=, CH=, and CH_2_ (30-H,36-H, 31-H, 35-H, 29-H, 34-H, and 27,28-H, 32,33-H, respectively). On the other hand, two of honokiol’s allyl groups are characterized by different environments, causing the protons 37,38-H, 35-H, 32,33-H to be downshifted compared to 29,30-H, 27-H, 24,25-H, respectively, as seen on the NOESY spectrum (Figure S2 given in the Supplementary Material). The NOESY spectrum for magnolol does not show a signal between 23-H and 26-H due to the molecule’s symmetry. Honokiol’s protons 24,25-H and 32,33-H can be distinguished based on ^13^C atoms connected to each proton on the HMBC spectrum. 31-C atom is coupled with 16-H proton only, while the 23-C atom couples with protons 9-H and 12-H (Figure S3 given in the Supplementary Material). Moreover, the non-Oppenheimer effect on honokiol’s NOESY spectrum can be observed between proton 12-H, which interacts with protons 22-H and 16-H. On the other hand, the position of 16-H gives only one signal interacting with proton 12-H, which allows us to distinguish it from proton 22-H (Figure S4 given in the Supplementary Material).

Quantitative comparison of spectra was conducted using principal components analysis (PCA) of the full spectra for magnolol and honokiol in four different solvents. It shows significant variation between spectra in terms of solvent. In the case of honokiol and magnolol, the 1D ^1^H spectra in CDCl_3_ and CD_2_Cl_2_ are grouped and differ significantly from spectra recorded in DMSO-d_6_ and acetone-d_6_ (Figure S5 given in the Supplementary Material).

**Figure 13:**
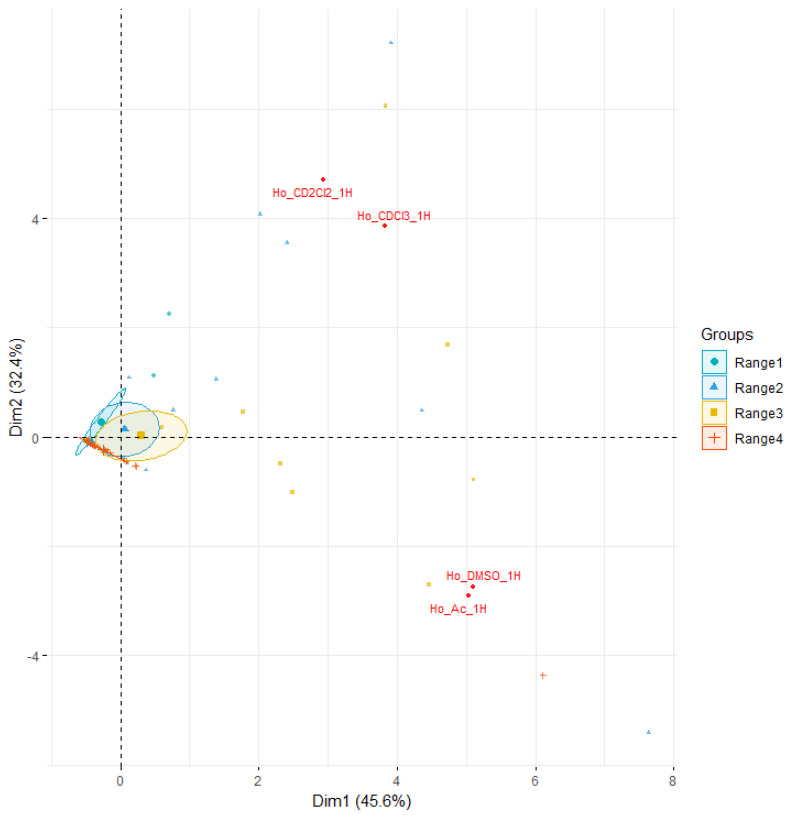

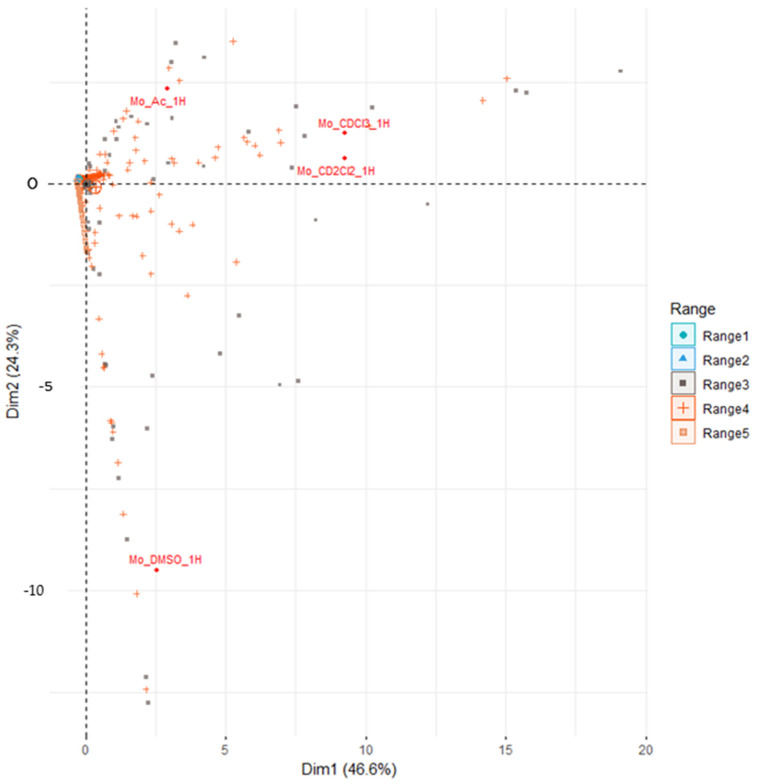
Biplot of honokiol (**up**) and magnolol (**down**) with respect to ^1^H regions. The variances of the full spectra are also placed on the spectra. The cycles points are on 0.95 confidence intervals.

We also analyzed the spectra more closely and found the regions that differ significantly in terms of different solvents. We divided the spectra of honokiol into four different regions, while magnolol required five different regions to cut all the solvents (Figure 13).

In the case of honokiol, the largest variance is observed in regions 2 and 3, while for magnolol, the largest variance can be observed in regions 4 and 5 (influenced mainly by the OH group; Figure 13).

Using DFT methods, we computed theoretical ^1^H NMR chemical shifts of magnolol **1** and honokiol **2** and compared the results with the experimental data (Table S4 given in the Supplementary Material). The computed and experimental values, along with the associated absolute and relative errors, are summarized in Table S4. The results indicate that the applied DFT protocol yields generally good agreement with the measured spectra. For magnolol, the calculated chemical shifts for aromatic protons (21H–26H) fall within the expected range (6.7–7.3 ppm) and closely match the experimental values, with errors typically below 0.2 ppm. The average mean absolute error (MAE) for all protons was calculated as 0.49 ppm, indicating satisfactory predictive accuracy for most non-exchangeable hydrogen atoms. However, significant deviations were observed for protons 37H and 38H, where computed shifts of 7.11 and 4.80 ppm differ markedly from experimental values of 9.08 ppm. These protons likely correspond to phenolic OH groups, whose observed shifts are strongly influenced by intermolecular hydrogen bonding and solvent exchange effects. A similar trend was observed for honokiol, where the aromatic protons (7H, 9H, 12H, etc.) are predicted with reasonable accuracy (errors < 0.2 ppm), and the calculated shifts for aliphatic or benzylic protons (24H, 25H, 33H) correlate well with experimental data. The average MAE for honokiol was slightly higher, at 0.70 ppm. The most pronounced discrepancies again correspond to exchangeable protons, namely 2H and 4H, where the computed values (4.23 and 6.00 ppm) significantly underestimate the experimental chemical shifts (9.19 and 9.33 ppm). These findings are consistent with prior reports highlighting the challenge of accurately modeling OH proton shifts without explicit solvent molecules or vibrational averaging procedures [36,37]. These results underline the general reliability of the DFT/GIAO method for predicting ^1^H NMR spectra of the analyzed phenolic compounds.

### 2.6. DSC Analysis

**Figure 14:**
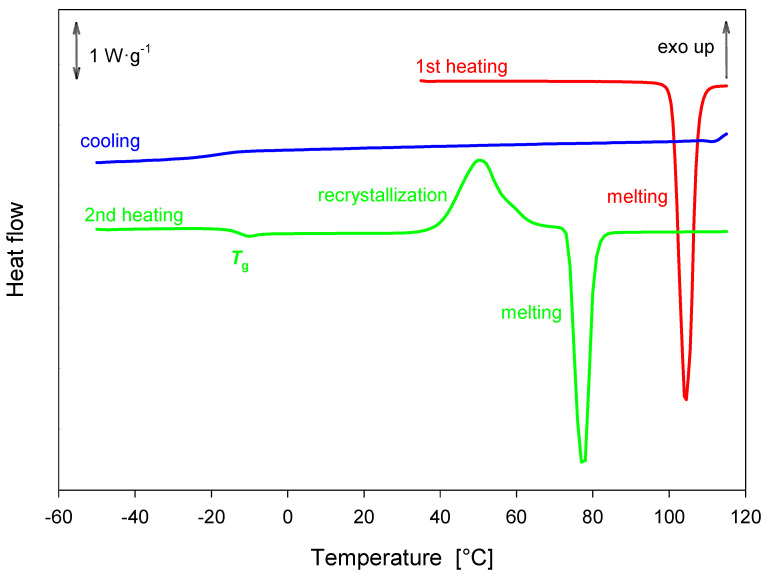
DSC analysis of magnolol obtained with a heating rate of 10 K·min^–1^.

During the first heating of the magnolol sample, a single melting event was observed at *T*_onset_ = 100.9 °C with an enthalpy of fusion Δ*h* = 134.30 ± 1.70 J·g^−1^. No recrystallization was observed during the cooling process. The glass transition temperature was estimated to be around *T*_g_^midpoint^ = −12.55 ± 0.33 °C with a heat capacity change of Δ*c_p_* = 0.38 ± 0.06 J·g^−1^·K^−1^. Recrystallization was observed prior to melting during the second heating of the sample. On the second heating, melting took place at *T*_onset_ = 72.53 ± 1.30 °C with Δ*h* = 94.46 ± 12,68 J·g^−1^, leading to the conclusion that magnolol exhibits two polymorphs with distinct melting temperatures. The described processes are shown in Figure 14.

**Figure 15:**
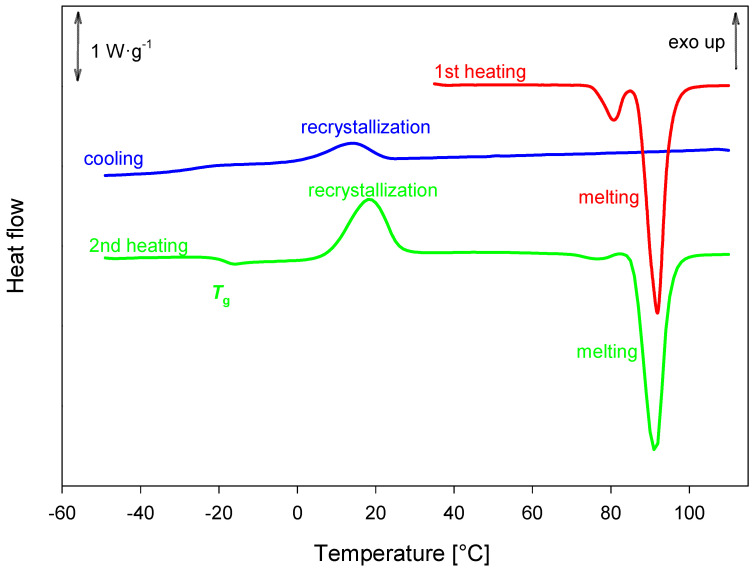
DSC analysis of honokiol obtained with a heating rate of 10 K·min^–1^.

During the initial heating of honokiol, two endothermic peaks were observed. One of these peaks, at *T*_onset_ = 75.7 ± 3.4 °C with Δ*h* = (13. ± 3J·g^−1^) likely corresponds to either melting or a polymorphic transition. Melting occurred at *T*_onset_ = (86.93 ± 37 °C) with Δ*h* = 99± 12J·g^−1^). Partial recrystallization was observed during the cooling process, resulting in a semicrystalline solid. The glass transition temperature was estimated to *T*_g_^midpoint^ = −20 ± 1 °C) with Δ*c_p_* = 0.43 ± 0,01 J·g^−1^·K^−1^. Further recrystallization was observed during the second heating. The described processes are shown in Figure 15.

**Figure 16:**
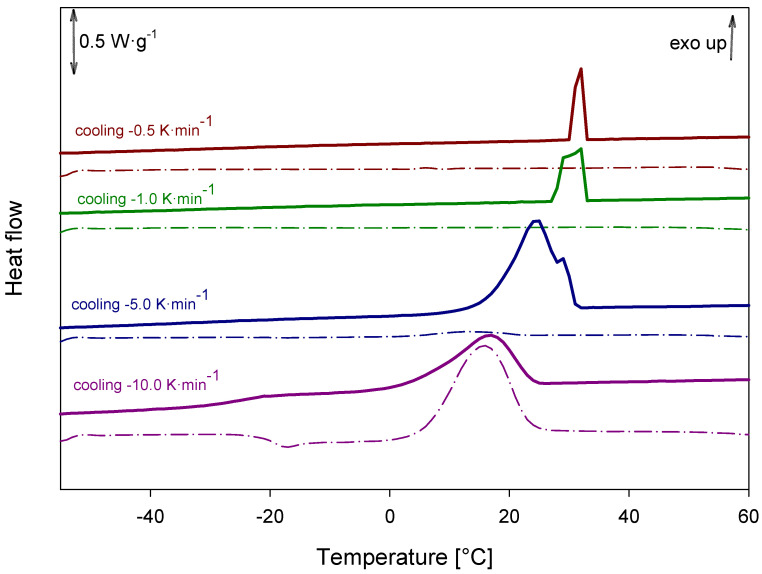
DSC analysis of honokiol after cooling at different rates.

Cooling honokiol samples at different cooling rates revealed that the rate of cooling significantly influenced the degree of recrystallization. Slower cooling promoted more extensive recrystallization, resulting in fully crystalline products, whereas faster cooling led to partial recrystallization and the formation of semicrystalline materials, as shown in Figure 16.

The TMDSC experiment was conducted with a heating rate of 3 K·min^−1^, ranging from 213.15 to 393.15 K (−60–120 °C, using a modulation amplitude of 1.5 K and a 60-s period with an underlying heating or cooling rate of 3 K·min^−1^. After reaching a temperature of 393.15 K (120 °C), the sample was subsequently cooled back down to 312.15 K (−60 °C). This entire experiment was repeated for 2.5 cycles. The glass transition temperature observed in the DSC reversing scheme was estimated to be approximately *T*_g_^midpoint^ = 256.4 ± 0.70 K (−16.75 ± 0.70 °C), which correlates with the standard DSC results.

## 3. Materials and Methods

### 3.1. Chemicals

Magnolol (**1**): 2-(2-Hydroxy-5-prop-2-enylphenyl)-4-prop-2-enylphenol, was purchased from Shouguang Fukang Pharmaceutical Co., Ltd., China, purity ≥ 99% (in compliance with European Pharmacopoeia 8.0).

Honokiol (**2**): 2-(4-Hydroxy-3-prop-2-enylphenyl)-4-prop-2-enylphenol, was purchased from Shouguang Fukang Pharmaceutical Co., Ltd., China, purity ≥ 99% (in compliance with European Pharmacopoeia 8.0).

### 3.2. Spectroscopy

The IR spectra were recorded in KBr (1.00 mg of compound **1** or **2** per 300 mg of KBr) on a Shimadzu IRAffinity-1 spectrometer.

The UV spectra were run on a Perkin Elmer UV/VIS Lambda 20 spectrophotometer in 1 cm quartz cuvettes using 0.004, 0.01, 0.02, 0.05 and 0.1 mg/mL solutions of compound **1** or 0.01, 0.02, 0.05, 0.1, 0.2, 0.5 and 1.0 mg/mL solutions of compound **2** in methanol.

The ^1^H NMR and ^13^C NMR spectra for magnolol and honokiol were acquired using a Bruker AVANCE II 400 MHz (9.39T) NMR spectrometer (^1^H) and/or a Bruker AVANCE III 500 MHz (11.74T) NMR spectrometer (^13^C). Magnolol or honokiol (10 mg) was dissolved in 500 μL of DMSO-d_6_ (Aldrich), CDCl_3_ (Aldrich), or acetone-d_6_ (Aldrich). TMS was used as an internal standard.

MS ESI spectra of **1** and **2** are given in the Supplementary Material (Figures S6 and S7). Mass spectrometry analysis was performed using a Q-Exactive Orbitrap mass spectrometer (Thermo Fisher Scientific, Bremen, Germany) equipped with a TriVersa NanoMate ESI ion source (Advion Biosciences Ltd., Ithaca, NY, USA) working in direct infusion mode. A total of 5 μL sample aliquots were infused directly into the mass spectrometer, and after ion current stabilization, spectra were acquired for 5 min. The Tri Versa source was operating at 1.25 psi nitrogen pressure, and the ionization voltage was set to 1.05 kV.

### 3.3. Theoretical Calculations

The initial structures of optimized rotamers of magnolol **1** and honokiol **2** were taken from the *.*cif* files given in the crystallographic base CCDC, and were named as follows: CIPXII [38] and WIKFIF02 [39], for **1** and **2**, respectively. They were initially optimized (*Gaussian 16 A.03* program [40]) using DFT formalism [41] namely: (*a*) B3LYP/6-31G(d,p) [42], (*b*) CAM-B3LYP/6-31G(d,p) [43], (*c*) B3LYP/6-311+G(d,p) [44,45], (*d*) PBE1PBE/6-31G(d,p) [46], (*e*) M06L/6-31G(d,p) [47], M062X/6-31G(d,p) [48], (*f*) APF/6-31G(d,p) [27], (*g*) APFD [27] and (*h*) PW6B95D3 [11] approaches in the gaseous phase (IR and UV spectrum calculations) or by applying the CPCM model [22] (UV and NMR spectrum calculations). Regarding the analysis of MEP for the charge distribution in the **1** and **2** optimized rotamers, we employed the keywords: “pop=full”, as well as the natural bond orbital (NBO, keywords: “pop=nbo”) as implemented in Gaussian G16 A.03 software, and the CHelpG (Charges from Electrostatic Potential) procedure with the calculation scheme developed by Breneman and Wiberg [22] (keyword “pop=chelpg”). In the latter scheme, atomic charges are fitted to reproduce the molecular electrostatic potential at several points around the molecule. The theoretical analysis of **1** and **2** IR spectra had been limited to the aforementioned *a*−*h* approaches without a correction term. We carried out the computations of **1** and **2** vibrational frequencies using the same level of theory as it was used for the SCF optimization procedure and Grimme’s D3 empirical (GD3) dispersion model [25] (for rotamers optimized using B3LYP, CAM-B3LYP, PB0, M06L, M062X, PW6B95D3 functionals; rotamer of **2** was previously optimized at the B3LYP/6-311+G(d,p) level of theory in gaseous phase) [26] and the Petersson–Frisch dispersion model from the APFD functional (for rotamer optimized using APF functional) [27]. For UV-Vis calculations, we applied the TD-DFT method [49], the CPCM solvation model, the linear response (LR) approach, and methanol as solvent. The Chemcraft 1.7 software was utilized for visualization of all optimized rotamers [50]. The HOMO–LUMO orbitals for compounds were generated based on checkpoint files using *GaussView 5.0* program [51]. The NMR shift for the TMS reference proton (Href = 31.6474 or 31.6474 for **1** or **2**, respectively) was calculated by B3LYP/6-31G(d,p) approach in DMSO at 293 K using the gauge-including atomic orbital (GIAO) method implemented in Gaussian G16 A.03 program and the protocol described in our previous report [19].

Principal component analysis (PCA) was conducted using the MestreNova software, version 15.1.0-38027. Prior to analysis, all spectra were appropriately phased, baseline-corrected, and aligned to ensure data quality. Solvent signals were excluded from the analysis to eliminate interference. The binning range was set to 0.02 ppm, normalization was carried out using the sum method, and scaling was applied using the Pareto approach.

### 3.4. DSC Experiments

Heat-flow curves were obtained using a DSC 214 Polyma (Erich NETZSCH GmbH & Co. Holding KG, Selb, Germany) under a nitrogen gas flow of 30 mL·min^–1^. In the typical experiment, sample powders (1–10 mg) were crimped in a *T*_zero_ standard aluminum pan with a puncture and heated at a 10 K·min^–1^ rate from 29.15 to 393.15 K (25–120 °C). The samples were then cooled at a rate of 10 K·min^–1^ to 213.15 K (–60 °C) and reheated to 393.15 K (120 °C) in a second run. The temperature and heat-flow were calibrated with indium (*T*_m_ = 156.65 °C, Δ_fus_*h* = 28.45 J·g^–1^). Each sample was tested at least three times. Melting and relaxation events are quoted as an onset temperature. The glass transition temperatures are quoted as midpoints. Additionally, honokiol cooling experiments were conducted at various cooling rates (0.5 K·min^–1^, 1 K·min^–1^, 5 K·min^–1^, 10 K·min^–1^) using the same equipment. All values were determined using Proteus Thermal Analysis v 8.0.3 (Erich NETZSCH GmbH & Co. Holding KG, Selb, Germany). The errors are quoted as one standard deviation.

## 4. Conclusions

Our study presents a comprehensive comparative analysis of magnolol and honokiol’s experimental and theoretical spectroscopic properties, focusing on FT-IR, UV-Vis, and ^1^H NMR spectra. The investigation employed multiple DFT functionals and basis sets to determine the most accurate computational approaches for reproducing experimental observations. For the FT-IR spectra, the B3LYP/6-311++G(d,p) functional yielded the best agreement with experimental data for both compounds, particularly for C–H, C=C, and C–O vibrations. Discrepancies were observed in the predicted O–H stretching bands, which appeared at higher wavenumbers in theoretical calculations, likely due to the gas-phase computational model. The UV-Vis spectral analysis confirmed that B3LYP/6-31++G(d,p) provided the closest match to experimental absorption maxima for magnolol, while wB97XD/6-311++G(2d,3p) was most suitable for honokiol. The observed solvent-dependent shifts (bathochromic in non-polar solvents like n-hexane and hypsochromic in polar solvents like methanol) highlighted the influence of solvent polarity on spectral properties, consistent with solvatochromic effects. TD-DFT calculations supported the experimental findings, indicating that the first electronic transitions in both compounds primarily involve HOMO–LUMO excitations localized on the biphenyl core. NMR analysis revealed significant solvent-dependent chemical shift variations, particularly for hydroxyl protons, which appeared only in DMSO-d_6_ due to slower exchange kinetics. The allyl and aromatic regions also showed distinct patterns, with magnolol exhibiting higher symmetry than honokiol. PCA of the full ^1^H spectra in different solvents demonstrated apparent clustering, reflecting solvent effects on chemical shift distributions. The differences in chemical shifts and coupling constants, particularly for hydroxyl and allylic protons, provided insight into the molecules’ structural symmetry and electronic environments. The NBO analysis revealed that the electron density distribution in both compounds is predominantly localized over the biphenyl cores, with strong σ-bonding interactions and hyperconjugation effects involving hydroxyl groups and aromatic systems. The HOMO–LUMO gap and related descriptors (chemical hardness, electronegativity, chemical potential) consistently showed higher values for magnolol across all functionals, indicating subtle differences in their electronic structures despite their isomeric relationship. Thermal analysis by DSC demonstrated distinct melting behaviors for the two compounds. Magnolol exhibited two polymorphic forms with different melting points (100.9 °C and 72.5 °C). At the same time, honokiol showed two endothermic events associated with melting and potential polymorphic transitions. Glass transition temperatures were also determined, with honokiol displaying lower Tg values than magnolol. The cooling rate significantly affected the recrystallization behavior of honokiol, highlighting its tendency to form semicrystalline or amorphous solids depending on processing conditions.

Overall, this work demonstrates that B3LYP/6-311++G(d,p) is a robust functional for reproducing the experimental FT-IR and UV-Vis spectra of magnolol, while wB97XD/6-311++G(2d,3p) is preferred for honokiol’s UV-Vis properties. The combined spectroscopic and theoretical analyses provide a detailed understanding of magnolol and honokiol’s structural, electronic, and conformational characteristics. These insights may support the design of future studies exploring their pharmacological potential and formulation strategies.

## Figures and Tables

**Table 1:** IR spectrum of compound **1**; B3LYP/6-311++G(d,p)//B3LYP/6-31G(d,p)/gas level of theory; υ: stretching; δ: in-plane bending; γ: out-of-plane bending.

	IR Spectrum of Magnolol	
Experimental Wavenumber (cm^−1^)	Vibrational Assignments	Calculated Wavenumber (cm^−1^)
		B3LYP/6-311++G(d,p)
ca 3170	ν OH	3863, 3728
3123, 3073	ν C-H arom	3222, 3208, 3202
3053, 2930, 2835	ν C-H alkyl	3249, 3247, 3197, 3189, 3175, 3165, 3155, 3082, 3079, 3040, 3037
1609, 1584	ν C-C arom	1674, 1668, 1652, 1635, 1543
1638	ν C=C	1731, 1730
1282, 1155	δ C-H arom	1278, 1268
1243	ν C-O asym	1253
1047, 1020, 947	ν C-O-C sym	1139, 1165
849, 823, 795	γ C-H	1032, 975, 953, 937, 867, 752

**Table 2:** IR spectrum of compound **2**; B3LYP/6-311++G(d,p)//B3LYP/6-31G(d,p)/gas level of theory; υ: stretching; δ: in-plane bending; γ: out-of-plane bending.

	IR Spectrum of Honokiol	
Experimental Wavenumber (cm^−1^)	Vibrational Assignments	Calculated Wavenumber (cm^−1^)
		B3LYP/6-311++G(d,p)
ca 3297	ν OH	3902, 3822
3260	ν C-H arom	3261, 3243, 3226, 3212, 3207, 3202
3083, 3054, 3000, 2977	ν C-H alkyl	3288, 3282, 3202, 3199, 3185, 3126, 3102, 3074, 3068
1611, 1586	ν C-C arom	1700, 1690
1636	ν C=C	1754, 1743
1325, 1276	δ C-H arom	1309, 1304, 1245
1217	ν C-O asym	1226
1052	ν C-O-C sym	1196, 1158
918, 822, 776	γ C-H	954, 944, 933, 685

**Table 3:** Several HOMO–LUMO descriptors calculated using the DFT formalism Koopman’s theorem; **APF**–APF/6-311++G(2d,3p)//APF/6-31G(d,p), **B3LYP**–B3LYP/6-311++G(2d,3p)//B3LYP/6-31G(d,p), **APFD**–APFD/6-311++G(2d,3p)//APFD/6-31G(d,p), **CAM**–CAM-B3LYP/6-311++G(2d,3p)//CAM-B3LYP/6-31G(d,p), **M06L**–M06L/6-311++G(2d,3p)//M06L/6-31G(d,p), **PBE1PBE**–PBE1PBE/6-311++G(2d,3p)//PBE1PBE/6-31G(d,p), **PW6B95D3**–PW6B95D3/6-311++G(2d,3p)//PW6B95D3/6-31G(d,p), **M062X**–M062X/6-311++G(2d,3p)//M062X/6-31G(d,p), **B3LYP_6311_Gdp–**B3LYP/6-311++G(2d,3p)//B3LYP/6-311++G(d,p), **wB97XD**–wB97XD/6-311++G(2d,3p)//wB97XD/6-31G(d,p).

Compound	Method	HOMO Energy	LUMO Energy	HOMO–LUMO Gap	First Ionization Potential	Electron Affinity	Chemical Potential	Chemical Hardness	Electronegativity
Magnolol	APFD	−6.0094	−0.9197	5.0896	6.0094	0.9197	−3.4646	2.5448	3.4646
	APF	−6.0072	−0.9279	5.0793	6.0072	0.9279	−3.4675	2.5396	3.4675
	B3LYP_6311Gdp	−5.9283	−0.9774	4.9508	5.9283	0.9774	−3.4529	2.4754	3.4529
	B3LYP	−5.8817	−1.0256	4.8561	5.8817	1.0256	−3.4537	2.4281	3.4537
	CAM-B3LYP	−7.2252	0.1913	7.4165	7.2252	−0.1913	−3.5169	3.7082	3.5169
	M062X	−7.1683	−0.2025	6.9658	7.1683	0.2025	−3.6854	3.4829	3.6854
	M06L	−5.1715	−1.4857	3.6858	5.1715	1.4857	−3.3286	1.8429	3.3286
	PBE1PBE	−6.0654	−0.8558	5.2096	6.0654	0.8558	−3.4606	2.6048	3.4606
	PW6B95D3	−6.1509	−0.7788	5.3721	6.1509	0.7788	−3.4648	2.6860	3.4648
	wB97XD	−7.7909	0.8411	8.6320	7.7909	−0.8411	−3.4749	4.3160	3.4749
Honokiol	APFD	−6.3144	−1.1549	5.1596	6.3144	1.1549	−3.7346	2.5798	3.7346
	APF	−6.3204	−1.1587	5.1617	6.3204	1.1587	−3.7395	2.5809	3.7395
	B3LYP_6311Gdp	−6.2216	−1.2079	5.0137	6.2216	1.2079	−3.7148	2.5069	3.7148
	B3LYP	−6.1925	−1.2446	4.9478	6.1925	1.2446	−3.7186	2.4739	3.7186
	CAM-B3LYP	−7.5716	−0.0291	7.5425	7.5716	0.0291	−3.8003	3.7712	3.8003
	M062X	−7.5267	−0.3899	7.1367	7.5267	0.3899	−3.9583	3.5684	3.9583
	M06L	−5.4540	−1.6757	3.7783	5.4540	1.6757	−3.5648	1.8892	3.5648
	PBE1PBE	−6.3843	−1.0841	5.3002	6.3843	1.0841	−3.7342	2.6501	3.7342
	PW6B95D3	−6.4673	−0.9834	5.4839	6.4673	0.9834	−3.7254	2.7420	3.7254
	wB97XD	−8.1169	0.6035	8.7204	8.1169	−0.6035	−3.7567	4.3602	3.7567

**Table 4:** Proton signals for experimental ^1^H NMR spectra of magnolol and honokiol (atom numbering as in Figure 1); DMSO as solvent.

Groups	Honokiol (Experimental Shift)	Magnolol (Experimental Shift)
OH	9.33 & 9.19 ppm	9.08 ppm, s
CH2–1	(24,25-H) 3.28 ppm, d, *J* = 6.7 Hz, 2H	(27,28,32,33-H) 3.31 ppm, d *J =* 6.8 Hz, 2H
CH2–2	(32,33-H) 3.37 ppm, d, *J* = 6.7 Hz, 2H	
CH–1	(27-H) 5.98–5.91 ppm	(29,34-H) 5.93–6.01 ppm
CH-2	(35-H) 5.99–6.06 ppm	
CH2vin–1	(37,38-H) 5.13–5.04 ppm, tq, broad,	(30,31,35,36-H) 5.02–5.11 ppm, m
CH2vin–2	(29,30-H) 5.04–4.99 ppm, m	
CHar	(20-H) 6.88–6.87 ppm(7-H) 6.87–6.86 ppm	(22,25-H) 6.98–7.00 ppm, (dd) *J* = 2.15*, J =* 8.20 Hz, 2H(21,24-H) 7.01–7.02 ppm, d *J* = 2.1 Hz*,* 2H
CHar	(9-H) 6.89–6.92 ppm, dd, *J* = 2.0 Hz, 8.2 Hz, 1H	-
CHar	(12-H) 7.02 ppm, d, *J* = 2.0 Hz, 1H	-
CHar	(22-H) 7.24–7.26 ppm, dd, *J* = 2.2, 8.2 Hz, 1H	(23,26-H) 6.89 ppm, d, *J* = 8.2 Hz, 2H
CHar	(16-H) 7.27–7.28 ppm, d, *J* = 2Hz, 1H	-

## Data Availability

Data are contained within the article and Supplementary Material.

## Supplementary Materials

The following supporting information can be downloaded at: https://www.mdpi.com/article/10.3390/ijms26136085/s1.

## Abbreviations

The following abbreviations are used in this manuscript:

FT-IR	Fourier-Transform Infrared Spectroscopy
UV-Vis	Ultraviolet–Visible Spectroscopy
NMR	Nuclear Magnetic Resonance
TD-DFT	Time-Dependent Density Functional Theory
DFT	Density Functional Theory
NBO	Natural Bond Orbital
PCA	Principal Component Analysis
HOMO	Highest Occupied Molecular Orbital
LUMO	Lowest Unoccupied Molecular Orbital
DSC	Differential Scanning Calorimetry
Tg	Glass Transition Temperature
∆Cp	Change in Heat Capacity
∆H	Enthalpy Change
BD*	Antibonding Orbital
RY*	Antibonding Rydberg Orbital
CIPXII	Crystallographic Identifier for Magnolol from CCDC
WIKFIF02	Crystallographic Identifier for Honokiol from CCDC
CCDC	Cambridge Crystallographic Data Centre
MS	Mass Spectrometry
CDCl3	Deuterated Chloroform (NMR solvent)
DMSO-d6	Deuterated Dimethyl Sulfoxide (NMR solvent)
CD2Cl2	Deuterated Dichloromethane (NMR solvent)
Tzero	Type of Standard Aluminum Pan (DSC equipment)
TMDSC	Temperature Modulated Differential Scanning Calorimetry
