# Supplementary material for: Structural and Spectroscopic Properties of Magnolol and Honokiol–Experimental and Theoretical Studies
Source: Int J Mol Sci. 2025 Jun 25;26(13):6085. doi: 10.3390/ijms26136085 (PMC12249702; doi:10.3390/ijms26136085)
Supplement: Supplementary file 1 [file ijms-26-06085-s001.zip › ijms-3703200-supplementary.pdf]

Structural and spectroscopic properties of Magnolol and Honokiol – Experimental and theoretical studies

Jacek Kujawski[1*], Beata Drabińska[1], Katarzyna Dettlaff[2], Marcin Skotnicki[3], Agata Olszewska[3], Tomasz Ratajczak[4], Marianna Napierała[1], Marcin K. Chmielewski[5], Milena Kasprzak[1], Kujawski Radosław[6], Aleksandra Gostyńska-Stawna[2], Maciej Stawny[2]

[1] Chair and Department of Organic Chemistry, Faculty of Pharmacy, Poznan University of Medical Sciences, Rokietnicka 3 str., 60-806 Poznań, Poland

[2]Chair Department of Pharmaceutical Chemistry, Faculty of Pharmacy, Poznan University of Medical Sciences, Rokietnicka 3 str., 60-806 Poznań, Poland

[3]Chair Department of Technology of Drugs, Faculty of Pharmacy, Poznan University of Medical Sciences, Rokietnicka 3 str., 60-806 Poznań, Poland

[4]Liquid Dosage Form Laboratory, Research and Development Department, Polpharma Warszawa S.A, Karolkowa 22/24 str., 01-207, Warsaw, Poland

5Institute of Bioorganic Chemistry, Polish Academy of Sciences, Noskowskiego 12/14 str.,
61-704 Poznań, Poland

[6]Chair Department of Pharmacology, Faculty of Pharmacy, Poznan University of Medical Sciences, Rokietnicka 3 str., 60-806 Poznań, Poland

[*]corresponding author: jacekkuj@ump.edu.pl; phone: +48616418505

Supplementary material

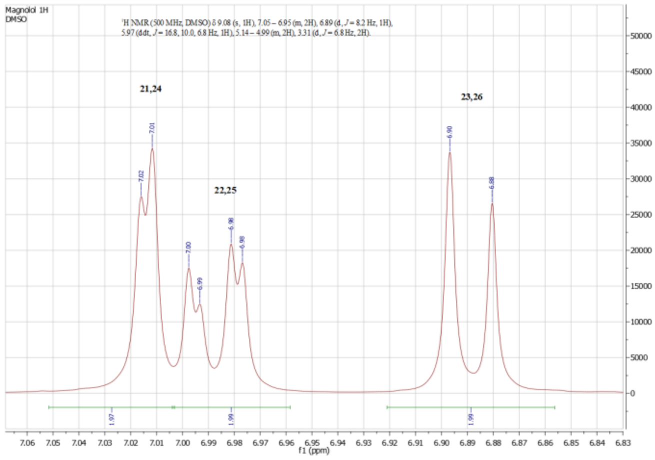

Figure S1. The ¹H spectrum of magnolol – aromatic region showing numeration and multiplets.

Honokiol – NOESY – allyl range	Magnolol - NOESY – allyl range
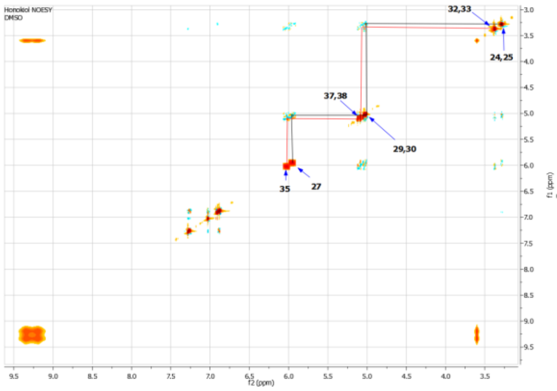	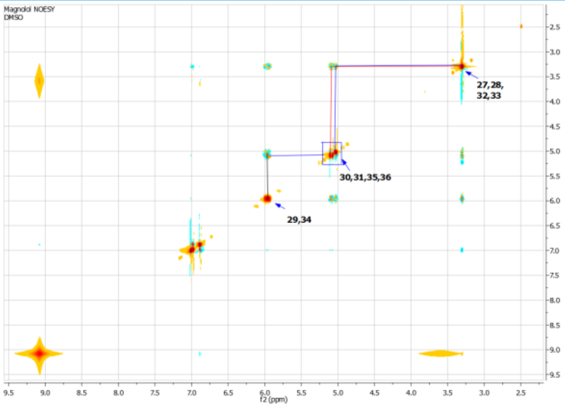

Figure S2. The NOESY spectra of magnolol and honokiol.

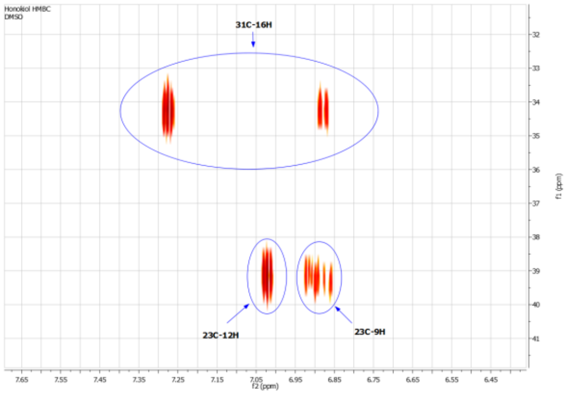

Figure S3. The HMBC spectrum of honokiol.

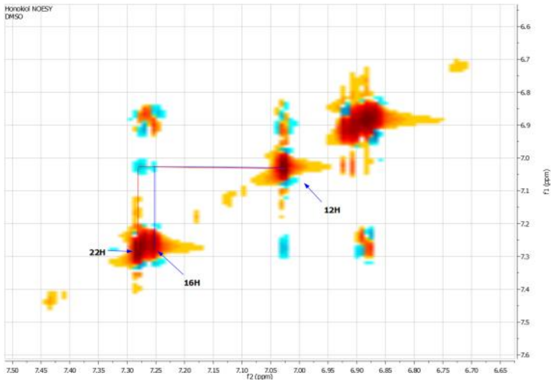

Figure S4. The NOESY spectrum of honokiol – aromatic region.

Magnolol – PCA - variables

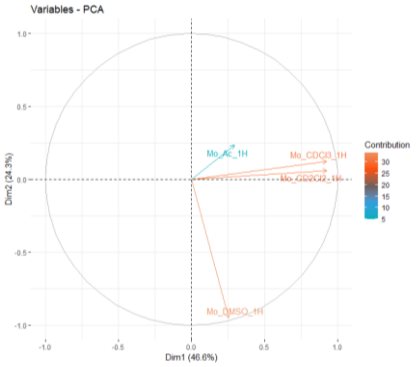

Honokiol – PCA - variables

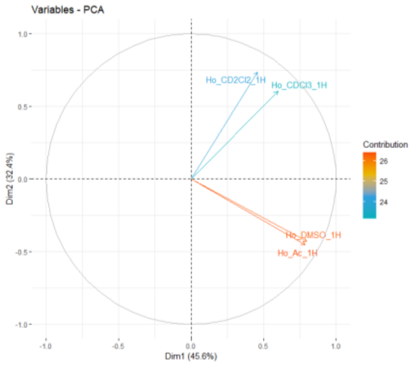

w~~W~~here~~:~~ Mo – Magnolol, Ho – Honokiol, Ac – acetone-d6, $CDCl_3$ – chloroform, CD_2Cl_2 – dichloromethane, DMSO – dimethylsulfoxide-d6

Figure S5. The plot of the PCA variables computed for magnolol and honokiol.

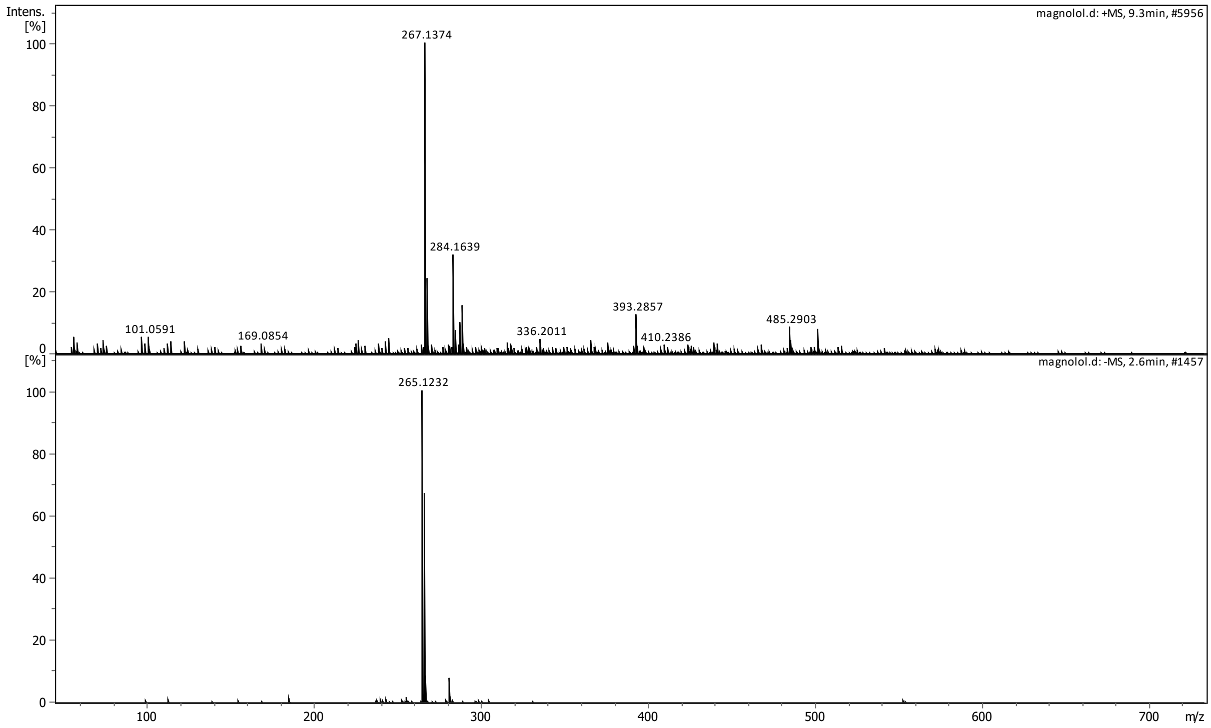

Figure S6. MS ESI spectra for magnolol.

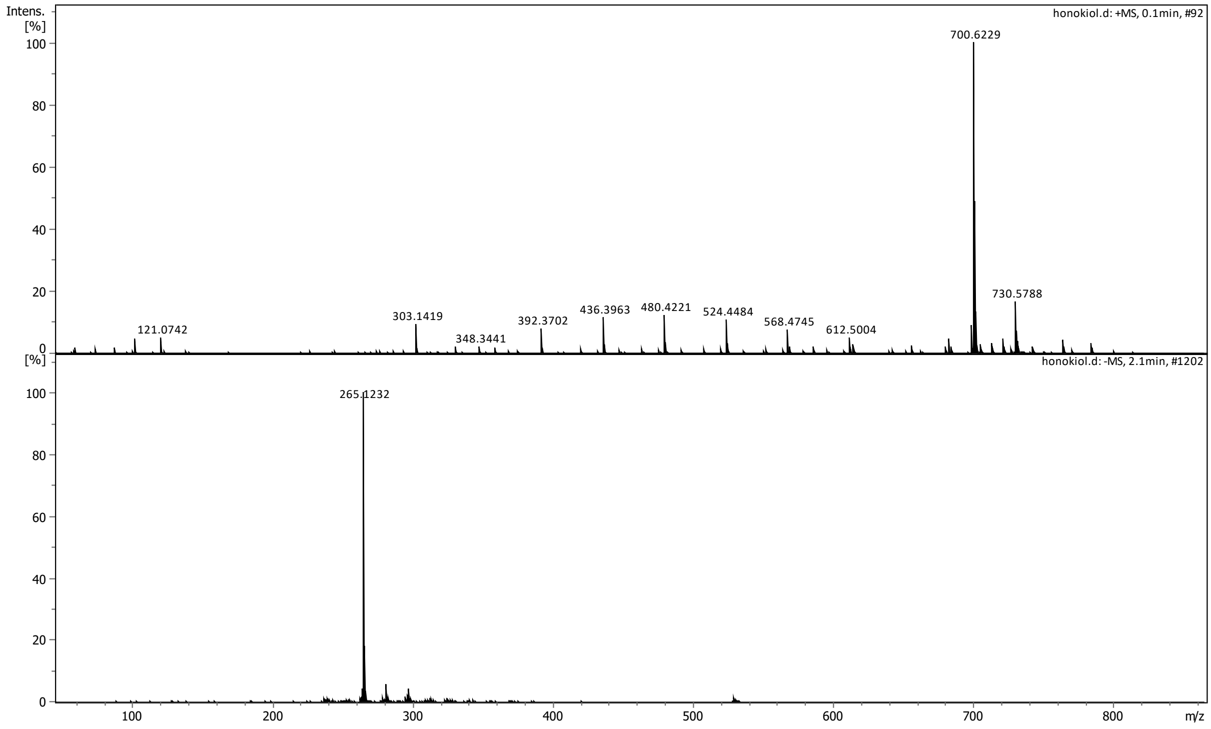

Figure S7. MS ESI spectra for honokiol.

Table S1. The atomic charges [e] for oxygen atoms within the structure of magnolol and honokiol (geometries optimized at the B3LYP/6-311+G(d,p) level of theory in gaseous phase, the Mulliken, CHelpG and NBO charges computations at the B3LYP/6-311++G(2d,3p) level of theory) estimated according to the Mulliken, CHelpG or NBO methodology (atoms numbering as in the Fig. 1).

**Heteroatom**	***Magnolol***		
	**Calculated charges**		
	**Mulliken**	**CHelpG**	**NBO**
**O5**	-0.242	-0.599	-0.707
**O6**	-0.128	-0.592	-0.709
**Heteroatom**	***Honokiol***		
	**Calculated charges**		
	**Mulliken**	**CHelpG**	**NBO**
**O1**	-0.560	-0.552	-0.681
**O3**	-0.624	-0.528	-0.696

Table S2. First excited states of the magnolol **1** computed using LR TD DFT approach in vacuum or in methanol; B3LYP/6-311++G(2d,3p)//B3LYP/6-311++G(d,p) level of theory.

**Compound 1**				
**Environ-ment**	**Energy [eV]**	**Wavelength [nm]**	**Oscillator strength**	**Ground state–first excit-ed state orbital transition**
Methanol	4.3844	282.79	0.2052	71 → 72

Table S3. First excited states of the honokiol **2** computed using LR TD DFT approach in vacuum or in methanol; wB97XD/6-311++G(2d,3p)//wB97XD/6-31G(d,p) level of theory.

**Compound 2**				
**Environ-ment**	**Energy [eV]**	**Wavelength [nm]**	**Oscillator strength**	**Ground state–first excit-ed state orbital transition**
Methanol	4.7471	261.18	0.3402	71 → 72

Table S4. Comparison of theoretical and experimental ^{1}H NMR shifts (atoms numbering as in ~~the~~ Fig. 1) for magnolol and honokiol.

Magnolol.

**Hydrogen**	**Theoretical (ppm)**	**Experimental (ppm)**	**Error (ppm)**	**Relative Error**
21H	7.22	7.01	-0.21	-0.03
22H	7.16	6.99	-0.17	-0.02
23H	6.84	6.89	0.05	0.01
24H	7.31	7.01	-0.30	-0.04
25H	7.14	6.99	-0.15	-0.02
26H	6.75	6.89	0.14	0.02
27H	3.39	3.31	-0.08	-0.02
28H	3.11	3.31	0.20	0.06
29H	6.06	5.97	-0.09	-0.02
30H	5.03	5.07	0.04	0.01
31H	5.35	5.07	-0.28	-0.05
32H	3.15	3.31	0.16	0.05
33H	3.38	3.31	-0.07	-0.02

34H	6.17	5.97	-0.20	-0.03
35H	5.20	5.07	-0.13	-0.03
36H	5.41	5.07	-0.34	-0.06
37H	7.11	9.08	1.97	0.28
38H	4.80	9.08	4.28	0.89

Honokiol.

Hydrogen	Theoretical (ppm)	Experimental (ppm)	Error (ppm)	Relative Error
2H	4.23	9.19	4.96	1.17
4H	6.00	9.33	3.33	0.56
7H	6.65	6.87	0.21	0.03
9H	7.03	6.91	-0.13	-0.02
12H	7.10	7.02	-0.08	-0.01
16H	7.09	7.28	0.19	0.03
20H	6.75	6.88	0.13	0.02
22H	7.76	7.25	-0.51	-0.07
24H	3.20	3.28	0.08	0.03
25H	3.17	3.28	0.11	0.03
27H	6.35	5.95	-0.41	-0.06
29H	4.52	5.01	0.49	0.11
30H	5.17	5.01	-0.16	-0.03
32H	3.63	3.37	-0.26	-0.07
33H	3.29	3.37	0.08	0.02
35H	6.39	6.03	-0.36	-0.06
37H	5.76	5.08	-0.67	-0.12
38H	5.45	5.08	-0.37	-0.07
